# Flagellin C decreases the expression of the *Gossypium hirsutum* cation/proton exchanger 3 gene to promote calcium ion, hydrogen peroxide, and nitric oxide and synergistically regulate the resistance of cotton to Verticillium wilt

**DOI:** 10.3389/fpls.2022.969506

**Published:** 2022-09-21

**Authors:** Heng Zhou, Yi Wang, Yihao Zhang, Yijing Xie, Hasan Nadeem, Canming Tang

**Affiliations:** ^1^State Key Laboratory of Crop Genetics and Germplasm Enhancement, College of Agriculture, Nanjing Agricultural University, Nanjing, China; ^2^State Key Laboratory of Cotton Biology, Institute of Cotton Research of Chinese Academy of Agricultural Sciences, Anyang, Henan, China

**Keywords:** flagellin, upland cotton, RNA-seq, immune response, *Verticillium dahliae*

## Abstract

To date, no ideal effective method for controlling Verticillium wilt in upland cotton (*Gossypium hirsutum*) has been defined. The purpose of this study was to determine the effects and mechanism through which flagellin C (*FLiC*) regulates the *Gossypium hirsutum* cation/proton exchanger 3 gene (*GhCAX3*), induces plant immunity, and increases resistance to Verticillium wilt. The FLiC gene was cloned from an endophytic bacterium (*Pseudomonas*) isolated from roots of the upland cotton cultivar Zhongmiansuo 41. The biocontrol effects of FLiC purified *in vitro* on resistant and susceptible upland cotton cultivars were 47.50 and 32.42%, respectively. FLiC induced a hypersensitive response (HR) in leaves of tobacco and immune responses in upland cotton. Transcriptome data showed that treatment with FLiC significantly enriched the calcium antiporter activity-associated disease-resistant metabolic pathway in seedlings. Moreover, FLiC downregulated *GhCAX3* expression to increase intracellular calcium ion (Ca^2+^) content and stimulate increases in the intracellular hydrogen peroxide (H_2_O_2_) and nitric oxide (NO) contents. The coordinated regulation of Ca^2+^, H_2_O_2_, and NO enhanced cotton resistance to Verticillium wilt. Furthermore, transgenic *Arabidopsis* plants overexpressing *FLiC* showed significantly improved resistance to Verticillium wilt. *FLiC* may be used as a resistance gene and a regulator to improve resistance to *Verticillium dahliae* (VD) in upland cotton.

## Highlights

– FLiC induces HRs in tobacco and immune responses in cotton.
– FLiC decreases *GhCAX3* expression to increase intracellular Ca^2+^content.
– Ca^2+^, H_2_O_2_, and NO synergistically regulate the resistance of cotton to VD.
– Transgenic Arabidopsis overexpressing *FLiC* shows significantly improved resistance to VD.

## Introduction

The immune response is closely related to disease resistance in plants. Plants rely mainly on defense pathways such as pathogenic microorganism pattern-triggered immunity (PTI; Nürnberger and Brunner, 2002) and pathogen-secreted effector-triggered immunity (ETI; Thomma et al., 2011; Naveed et al., 2020) to resist pathogen infection. The defense response is achieved by the mutual recognition of and interaction between the recognition receptors of plant cells and the elicitors secreted by pathogenic microorganisms. Systemic disease resistance in plants is ultimately achieved through the transmission and transduction of a series of signals that activate the immune response (Bouizgarne et al., 2006; Jones and Dangl, 2006; Kumar et al., 2020). The early defense response of plants mainly includes the production of early disease resistance signals, such as intracellular calcium ion (Ca^2+^) influx, reactive oxygen species (ROS) bursts, and nitric oxide (NO) accumulation (Marcec et al., 2019). Ca^2+^ is a key second messenger and mediates the response of plants to biotic and abiotic stimuli (Gao et al., 2021; Köster et al., 2022). Spatially and temporally defined cytoplasmic Ca^2+^ signals are shaped through the concerted activity of ion channels, exchangers, and pumps in response to diverse stimuli, and these signals are then decoded through the activity of Ca^2+^ binding sensor proteins (Köster et al., 2022). In plants, Ca^2+^ signaling is central to both pattern- and effector-triggered immunity, and the generation of characteristic cytoplasmic Ca^2+^ elevations in response to potential pathogens are common to both immune reactions (Köster et al., 2022). ROS are widely produced in different cellular compartments under both biotic and abiotic stress conditions (Qi et al., 2017). The plant perception of pathogen-associated molecular patterns triggers a plethora of cellular immune responses, and one of these responses is a rapid and transient burst of ROS mediated by plasma membrane-localized Nicotinamide adenine dinucleotide phosphate (NADPH) oxidases (Sang and Macho, 2017). Numerous studies have demonstrated the importance of NO in the regulation of plant defense against fungal pathogens. NO triggers a reprogramming of defense-related gene expression, the production of secondary metabolites with antimicrobial properties, and the hypersensitive response (HR; Martínez-Medina et al., 2019). These stimulus signals are converted from extracellular signals to intracellular signals and amplified through a cascade reaction to induce downstream defense reactions. These reactions always first occur around the infected tissue and then gradually spread to the surrounding uninfected tissues. The immune system of the entire plant is activated to defend against infection by various pathogens (Dixon et al., 1994; Yano et al., 1998; Buxdorf et al., 2013; Holmes et al., 2021). The resistance of plants is related to NO, hydrogen peroxide (H_2_O_2_), and Ca^2+^. The silencing of the Respiratory Burst Oxidase Homolog Protein D (GhRbohD) genes damage the resistance of cotton to fungi, weaken the generation of ROS, and decrease the content of NO, H_2_O_2_, and Ca^2+^ (Huang et al., 2021). However, few studies have investigated the mutual regulation of Ca^2+^, H_2_O_2_, and NO to mediate the resistance of cotton to Verticillium wilt.

Flagellin isolated from bacteria induces immune responses in arabidopsis, rice, algae, and kelp, but the underlying mechanisms are unclear (Takai et al., 2008; Wang, 2012; Wang et al., 2013; Ma et al., 2017). Plants stimulated by flagellin produce a series of defense responses, including ethylene (ETH) production, callose deposition, ROS bursts, defense-related gene expression, and growth inhibition (Asai et al., 2002; Zipfel et al., 2004). Calcium transporter-related regulatory genes positively and negatively regulate the intracellular Ca^2+^ levels to participate in plant defense responses (Bi et al., 2021; Liu et al., 2021). Plant Ca^2+^ gradients, which are found at millimolar levels in the vacuole and molar levels in the cytoplasm, are regulated in part by high-capacity vacuolar cation/H(+) exchangers (Manohar et al., 2011). Therefore, Ca^2+^ channels are crucial in maintaining Ca^2+^ levels and regulating plant immune responses (Thor et al., 2020). Cation/proton exchanger 3 (CAX3), a calcium transporter-related regulatory gene, can regulate the Ca^2+^ levels (Hocking et al., 2017; Li et al., 2021). The mechanism through which flagellin regulates *Gossypium hirsutum* cation/proton exchanger 3 (*GhCAX3*) in cotton to induce the plant immune response has not yet been reported. Verticillium wilt caused by *Verticillium dahliae* (VD) is the most devastating disease of cotton worldwide. No effective measure for controlling Verticillium wilt in upland cotton has been found. This study provides the first demonstration of the effects of flagellin C (FLiC) and the mechanism through which FLiC induces an immune response in upland cotton to increase resistance against VD. In this study, *FLiC* was cloned from an upland cotton endophytic bacterium (*Pseudomonas aeruginosa*) and purified *in vitro*, and the effects of FLiC and the mechanism through which FLiC regulates *GhCAX3* to enhance cotton immunity and increase resistance to VD were explored.

## Materials and methods

### Microbial strains

VD V1070 was generously provided by the Institute of Plant Protection, Jiansu Academy of Agriculture Sciences. Hygromycin B-resistant GFP-labeled VD was maintained on potato dextrose agar (PDA) culture medium at 25°C (Hu, 2012).

### Plant materials, culture conditions, and treatments

Seeds of upland cotton (*Gossypium hirsutum*) cultivars Zhongzhimian 2 and Jimian 11 and *Nicotiana benthamiana* were sown in soil, and the seedlings were grown in a greenhouse under an 8-h dark/16-h light photoperiod at temperatures of 23°C (dark)/28°C (light) with 60% relative humidity. Seeds of *Arabidopsis thaliana* were sterilized in 75% ethanol and 3% NaClO for 30 min and then washed three times with sterilized water. The Arabidopsis seeds were sown on 1/2-strength Murashige and Skoog (MS) medium containing 3% sucrose and 0.8% (w/v) agar for 2 weeks. Once they reached the three-leaf stage, the Arabidopsis seedlings were transplanted into nutrient-enriched soil and cultivated in a growth chamber at 22/20°C (night/day) with 60% relative humidity and a 16-h day/8-h night photoperiod. The roots of cotton and *Arabidopsis thaliana* plants were inoculated *via* irrigation with spore suspensions adjusted to a concentration of 1 × 107 spores/ml (Feng et al., 2021).

### Prokaryotic expression and purification of the FLiC protein

A PGEX-4 T-2 expression vector purchased from Beijing Kinco Xinye Biotechnology Co., Ltd., was used, and a pair of specific primers (*FLiC*-Pgex-4 T-2F/R) were designed based on the *FLiC* gene sequence, which is shown in Supplementary Table S2. The recombinant expression plasmid *FLiC*-PGEX-4 T-2 (verified by PCR amplification, restriction enzyme digestion, and sequencing) was transformed into competent *Escherichia coli* BL21 (DE3) cells for prokaryotic expression. A single colony of the positive strain was selected, inoculated into 5 ml of fresh LB liquid medium supplemented with 50 mg/l ampicillin, and cultured with shaking at 37°C until the OD_600_ reached 0.6–0.8. Isopropyl-β-D thiogalactopyranoside (IPTG) was added to a final concentration of 0.5 mM, and the culture solution was shaken at 150 rpm for 5 h at 28°C. FLiC protein was purified using a Beyotime GST-tagged Protein Purification Kit (Shanghai, China). FLiC protein was detected by sodium dodecyl sulfate–polyacrylamide gel electrophoresis (SDS–PAGE; 12.5%), and liquid that was not inoculated with IPTG was used as a control.

### FLiC protein HR assays

Tobacco (*Nicotiana benthamiana*) leaves were used as the experimental materials; 50 μl of FLiC protein solution (100 μg/ml) was injected into the mesophyll from the abaxial side of the leaves, and H_2_O was used as a control (Felix et al., 1999; Gómez-Gómez and Boller, 2000). Each experiment was repeated three times.

### Plant disease resistance assays

Four-leaf stage seedlings of uniformly growing disease-resistant upland cotton varieties (Zhongzhimian 2) and susceptible varieties (Jimian 11) were selected, and FLiC at a concentration of 100 μg/ml was uniformly applied to the upland cotton leaves; H_2_O was used as a control. Two days after being sprayed with the FLiC solution, the seedlings were inoculated with a pathogen spore suspension (2 × 10^7^ CFU/ml) using the following method: the upland cotton seedlings were removed, the roots of the experimental plants were soaked in the pathogen spore suspension for 20 min, and the control roots was soaked with H_2_O. After 15 days, the roots, stems and leaves of the upland cotton plants infected with VD were observed under a microscope (MVX10 MacroView, OLYMPUS, Japan). After 30 days, the disease index of Verticillium wilt was investigated. The disease severity of the seedlings was scored at 30 dpi according to the following criteria: 0 = healthy with no symptoms on the leaves; 1 = one or two cotyledons showing symptoms; 2 = a single true leaf showing symptoms; 3 = more than two leaves showing symptoms; and 4 = plant death. The overall disease index and control efficacy were calculated as follows (Supplementary Figure S1; Liu et al., 2014):


$$\mathrm{Disease index}=[\left({\mathrm{0n}}_{0}+{\mathrm{1n}}_{1}+{\mathrm{2n}}_{2}+{\mathrm{3n}}_{3}+{\mathrm{4n}}_{4}\right)/\mathrm{4n]}\times 100$$

$$\begin{array}{l} Efficacy\;\left(\%\right)=[Diseaseindex\;\left(control\right)-Diseaseindex\\ \left(treatment\right)]/Diseaseindex\;\left(control\right)\times 100 \end{array}$$

where *n*_0_–*n*_4_ are the numbers of plants with each of the corresponding disease scores and *n* is the total number of plants assessed. All experiments were repeated three times, and more than 80 plants were counted each time.

### Lignin and callose detection

Forty-eight hours after the cotton stems were sprayed with the FLiC solution, the stems of each group were randomly selected to detect the content of lignin according to the method descried by Xiong et al. (2021). The total lignin content of stem tissue was determined by the acetyl bromide method. Stem samples (100 mg) were rapidly ground into powder in liquid nitrogen, and then washed sequentially with ethanol, 95% ethanol, and deionized water to remove soluble substances. After lyophilization, add 5 ml of acetyl bromide-glacial acetic acid (1:3, vol/vol) to each sample and incubate at 70°C for 30 min. Then 0.9 ml of 2 M NaOH and 3 ml of glacial acetic acid were added, and finally 1 ml of 7.5 M hydroxylamine hydrochloride solution was added to stop the reaction. After centrifugation at 13,000 rpm, the supernatant was collected and the absorbance at 280 nm was measured with a microplate reader SpectraMax iD5. Alkali lignin was used as the standard curve (y = 0.969x + 0.009, *R*^2^ = 0.9978). The method described by Millet et al. (2010) was used for callose detection. The amount of callose was quantified using ImageJ software (version 1.48). Three independent biological and technical repeats were performed.

### VD biomass detection

Two cotyledons were removed from the cotton seedlings of the treatment groups in a sterile environment, weighed and added to 2 ml of sterile water for grinding. After ground into a homogenous mixture, the mixture was diluted according to the gradient dilution method. One hundred microliters of the diluent at different concentrations was spread on red Bengal-resistant medium supplemented with 50 μg/ml hygromycin and 50 μg/ml streptomycin and then placed in an incubator with a constant temperature of 28°C and cultured upside down for 2 days. Afterward, the number of colonies in each Petri dish was counted, and the content of VD per gram of leaf was calculated (Wang, 2014). Three independent biological and technical repeats were performed.

### Measurement of chitinase, glucanase, phenylalanine ammonia lyase, polyphenol oxidase, peroxidase, and catalase activities

Chitinase activity was measured using the method described by Emani et al. (2003), and GLU activity was measured using the procedure descried by Abeles and Forrence (1970). PAL activity was measured using the method described by Dickerson et al. (1984), and PPO activity was measured as described by Dickerson et al. (1984). POD activity was measured using the method described by Dong et al. (2003), and CAT activity was measured as described by Plazek and Zur (2003). Three independent biological and technical repeats were performed.

### Measurement of resistance-related gene expression by quantitative reverse transcription PCR

Cotton cotyledons were collected, and quantitative reverse transcription PCR (qRT–PCR) analysis was conducted at different time points. RNA extraction and gene expression analysis were performed as previously described (Ren et al., 2013). Each real-time assay was tested *via* a dissociation protocol to ensure that each amplicon comprised a single product. Sequences of upland cotton defense-related gene primers were used for qRT–PCR (Supplementary Table S1; Han, 2014). The relative expression of genes was calculated using the 2^-ΔΔCT^ method (Livak and Schmittgen, 2001). Technical replicates of three independent biological samples were performed.

### Ca^2+^, H_2_O_2_, and NO detection and quantification

Twelve hours after being sprayed with FLiC protein solution, leaves of each group of plants were randomly selected, and the Ca^2+^ fluorescence was measured using the method described by Chen et al. (2004). A Calcium Colorimetric Assay Kit (Beyotime Biotechnology Company, Shanghai, China) was used to quantify the Ca^2+^ concentration. The images were analyzed using Leica Image software. Twelve hours after being sprayed with FLiC protein solution, leaves of each group of plants were randomly selected and stained with Diaminobenzidine (DAB) staining solution. The H_2_O_2_ level was measured as described by Kumar et al. (2009). Cotton leaves were washed with distilled water, placed in a Petri dish, mixed with an appropriate amount of DAB (1 mg/ml), and stained under light at 25°C for 8 h. After the staining solution was removed, 95% ethanol solution was added to remove the chlorophyll in the leaves. After boiling for 20 min, the green leaves were removed. The H_2_O_2_ deposition in leaves was observed after suspension in clear water. A Hydrogen Peroxide Assay Kit (Beyotime Biotechnology Company, Shanghai, China) was used to quantify the H_2_O_2_ concentration, and the NO concentration was measured using the method described by Sun et al. (2012). All the images were visualized by confocal laser scanning microscopy (CLSM; excitation 488 nm; emission 515 nm) and analyzed using Leica Image software. Three independent biological and technical repeats were performed.

### RNA sequencing

Two-leaf stage Zhongzhimian 2 plants were used as the materials for transcriptome sequencing. Leaves sprayed with flagellin FLiC were used as the treatment group, and leaves sprayed with water served as the control group. Three replicates of the treatment group and the control group were included, resulting in a total of six transcriptome sequencing samples. Cotton total RNA was extracted with the E.Z.N.A. TM Plant RNA Mini Kit (WEGENE, Shanghai, China). The mRNA with a polyA structure in total RNA was enriched by Oligo(dT) magnetic beads, and the RNA was broken into fragments using the ion breaking method. First-strand cDNA was synthesized using RNA as the template, six-base random primers and reverse transcriptase, and second-strand cDNA was synthesized using first-strand cDNA as the template. After construction of the library, the library fragments were enriched by PCR amplification. The quality of the library was checked using the Agilent 2100 Bioanalyzer, and the total and effective concentrations of the library were then detected. Libraries containing different index sequences were then mixed in proportions based on the effective concentration of the library and the amount of data needed for the library. The pooled libraries were uniformly diluted to 2 nM, and single-stranded libraries were formed by alkali denaturation. After RNA extraction, purification, and library construction, the libraries were paired-end (PE) sequenced based on the Illumina sequencing platform. Based on the RNA sequencing (RNA-seq) results, 12 genes were randomly selected for the design of gene-specific primers using information within the NCBI database and for verification by qRT–PCR (Supplementary Table S2).

### GO and KEGG pathway enrichment

Based on the transcriptome screening data, genes that met the following conditions were considered to be differentially expressed: log2|fold change| ≥ 1 and a value of *p* ≤ 0.05. Gene Ontology (GO) terms and Kyoto Encyclopedia of Genes and Genomes (KEGG) pathways were considered to be significantly enriched in the differentially expressed genes (DEGs) if the *p* values were <0.05. GO enrichment analysis maps the differentially expressed genes to each term in the database, the number of DEGs in each term is counted, and the GO terms with the same genetic background as the sample DEGs are selected. The DEGs were functionally classified according to the GO annotation results. The enrichment analysis was performed using the phyper function in R software. Biological pathway classification of DEGs was performed based on the KEGG annotation results. Significant enrichment analysis was performed to analyze the physiological pathways and signal transduction pathways involving the DEGs.

### Virus-induced gene silencing

The fragments containing part of the coding sequence of *GhCAX3* were amplified from the cDNA of Zhongzhimian 2 using the corresponding primer pair *GhCAX3*-VIGS-F/R and then cloned into a TRV:00 plasmid at the XbaI BamHI sites *via* T4 DNA Ligase (TaKaRa). The TRV vectors were then transformed into *Agrobacterium tumefaciens* GV3101. The transformed TRV vectors were agroinfiltrated into two fully expanded cotyledons of Zhongzhimian 2 cotton seedlings. Afterward, the transformed seedlings were transferred to a growth chamber at 25°C with a 16-h light/8-h dark photoperiod. At least 60 plants were inoculated with each construct. A TRV:CLA construct served as a positive marker for evaluating the VIGS efficiency. Fifteen days after inoculation, the expression levels of the target gene were examined, and the successfully silenced plants were subjected to infection with VD V1070. The primers used for this experiment are listed in Supplementary Table S2.

### Arabidopsis transformation

The open reading frame (ORF) of FLiC was cloned and inserted into a pBI121 (Cambia) plant binary vector together with the 35S promoter. The resulting pBI121-FLiC plasmid was introduced into *Agrobacterium tumefaciens* strain GV3101. The primers (pBI121-FLiC F/R) used are listed in Supplementary Table S2. The transformants (T0, T1, and T2 seeds) were screened for survival on half-strength MS medium supplemented with 50 mg/L kanamycin. T3 transgenic lines were identified by RT–PCR.

### Statistical analysis

All the experiments used to obtain the data described in this paper were repeated at least three times. The resulting data were subjected to ANOVA using SPSS 19.0 software (SPSS, Inc.). Student’s *t*-test and one-way ANOVA (*p* < 0.05) followed by Duncan tests were used for multiple comparisons.

## Results

### Structure and HR effects of FLiC

**Figure 1:**
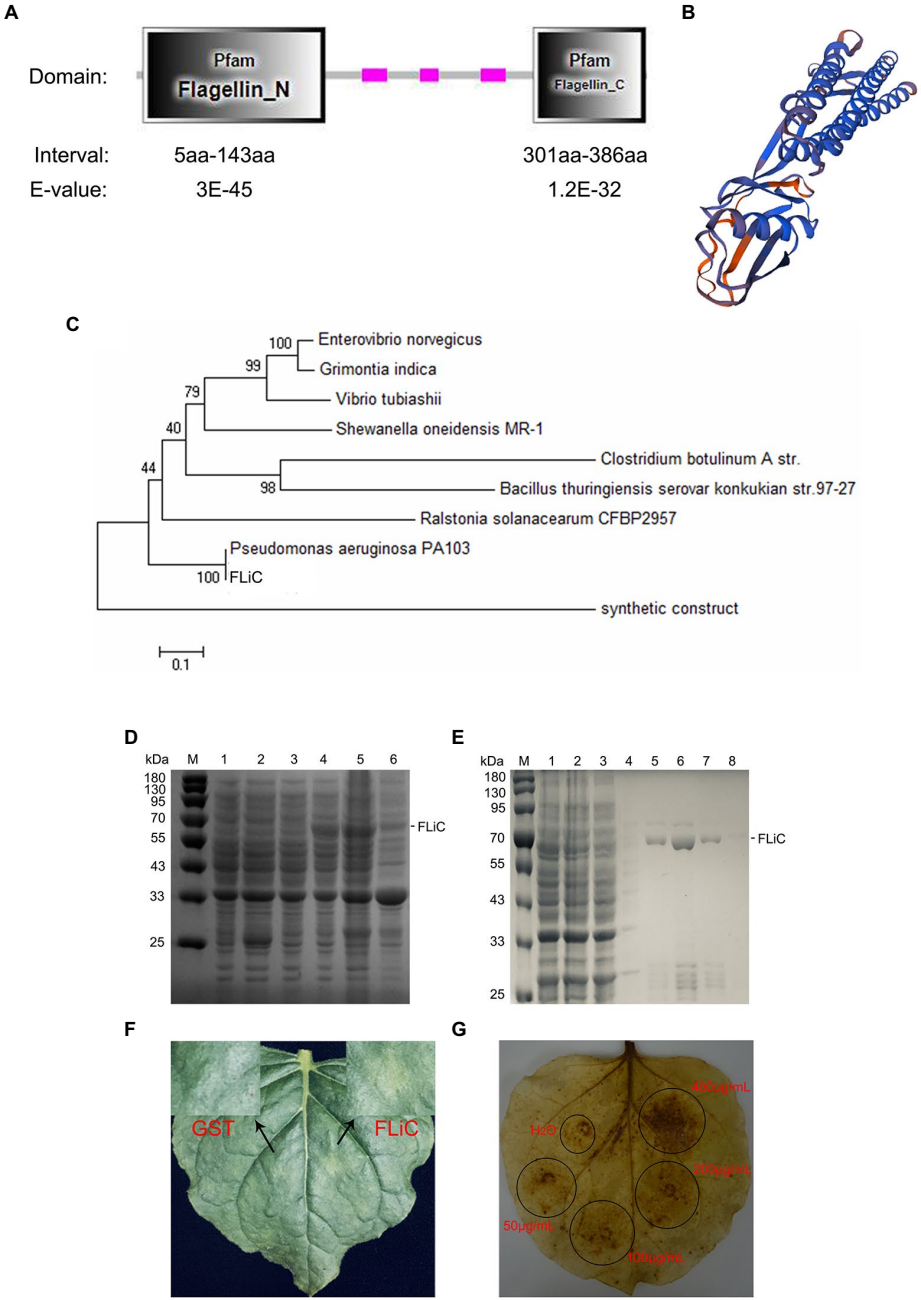
Structure and hypersensitive response (HR) of flagellin C (FLiC). **(A)** Two functional domains of FLiC protein. **(B)** Three-dimensional structure diagram of FLiC protein. **(C)** Evolutionary tree of FLiC proteins. **(D)** Expression of FLiC recombinant protein in *Escherichia coli* at 28°C [M protein marker; (1) control containing an empty vector; (2) control containing an empty vector with addition of an inducer (0.5 mM IPTG); (3) whole bacteria with addition of an inducer (0 mM IPTG); (4) whole bacteria with addition of an inducer (0.5 mM IPTG); (5) supernatant after the bacterial cells were sheared; (6) pellet after the bacterial cells were sheared]. **(E)** Purified FLiC protein (M protein marker; 1, cell lysate; 2, flow-through; 3, wash 1; 4, wash 2; 5, elution 1; 6, elution 2; 7, elution 3; and 8, elution 4). **(F)** HR of tobacco treated with FLiC. The concentration of FLiC protein was 100 μg/ml; the photograph shows the backside of tobacco 24 h after injection. **(G)** Hydrogen peroxide (H_2_O_2_) generated after treatment with FLiC protein solutions at different concentrations. Diaminobenzidine (DAB) staining was used to visualize H_2_O_2_.

The *FLiC* gene was cloned from an endophytic bacterium (*Pseudomonas aeruginosa*) isolated from the roots of the upland cotton cultivar Zhongmiansuo 41. The FLiC protein is a type of flagellin with two functional domains (Figure 1A); its three-dimensional structure is shown in Figure 1B. FLiC exhibits 100% homology with *Pseudomonas aeruginosa* PA103 (Figure 1C). The recombinant plasmid was transformed into *Escherichia coli* strain BL21 (DE3) and induced by IPTG (0.5 mM) at 28°C for 5 h. The purified FLiC protein was 66 kDa (Figures 1D,E) and induced HR effects and an ROS burst in tobacco leaves (Figures 1F,G).

### FLiC induces resistance to VD in upland cotton plants

**Figure 2:**
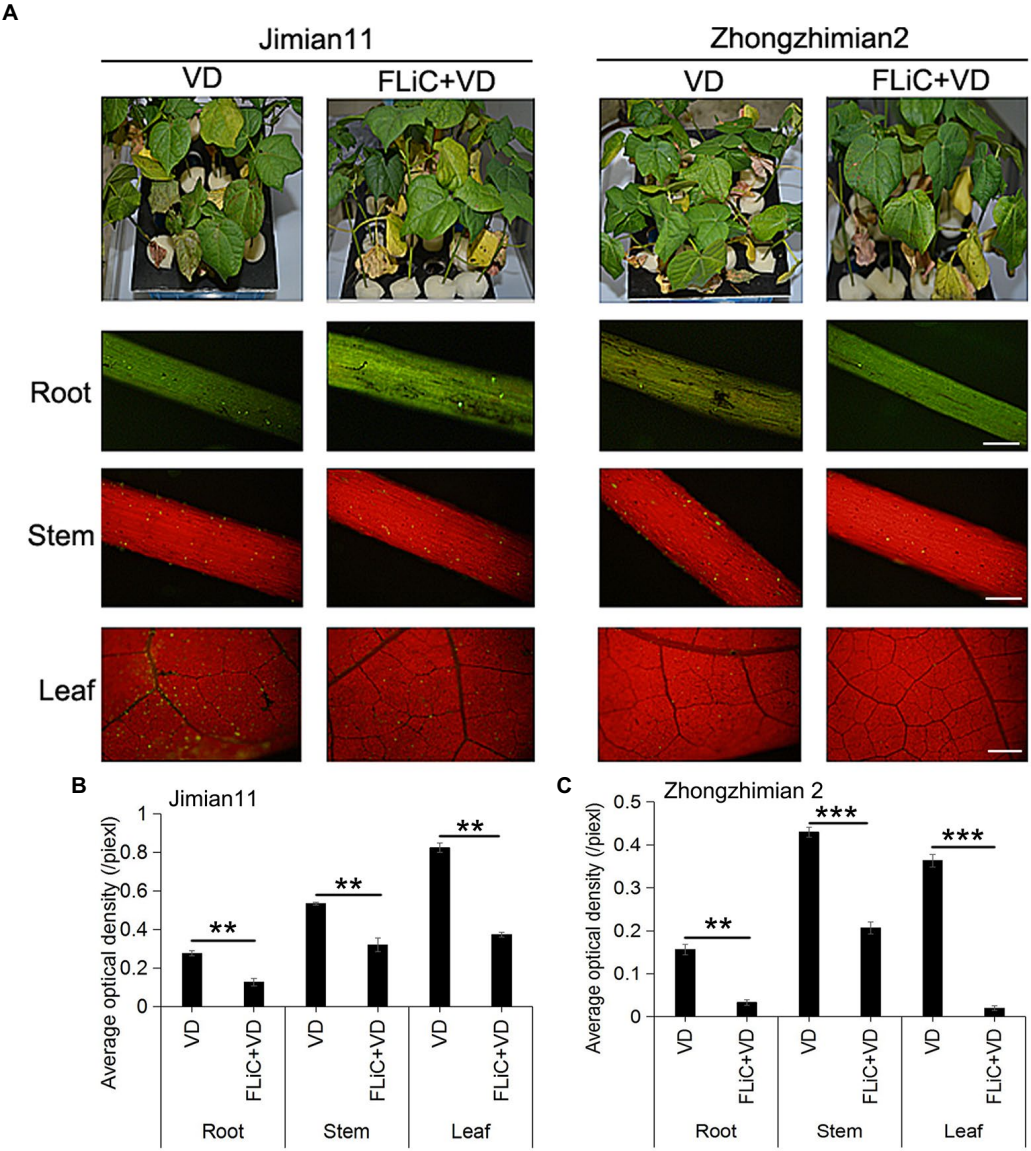
Effect of FLiC on infection with VD in Jimian 11 and Zhongzhimian 2. **(A)** Differences in phenotype and spore colonization of the roots, stems, and leaves between the disease-resistant variety Zhongzhimian 2 and the susceptible variety Jimian 11. **(B)** Statistics of the average fluorescence density of spores in the roots, stems, and leaves of Jimian 11. **(C)** Statistics of the average fluorescence density of spores in the roots, stems, and leaves of Zhongzhimian 2. The data represent the means ± SDs; *n* = 3 (**p < 0.01; ***p < 0.001; *t* test).Bars = 500 μm.

Jimian 11 (a susceptible cultivar) and Zhongzhimian 2 (a resistant cultivar) were sprayed with FLiC protein solution, and the amount of VD infection in the roots, stems, and leaves of these plants were significantly lower than those found in the control group under the microscope (Figures 2B,C), indicating that FLiC can induce resistance to VD infection (Figure 2). Moreover, according to the classification standard of cotton verticillium wilt disease (Supplementary Figure S1), the incidence of infection in Zhongzhimian 2 was lower than that in Jimian 11. Thirty days after FLiC treatment, the disease index of Zhongzhimian 2 (29) was lower than that of Jimian 11 (46.23). The relative biocontrol effects of FLiC on Zhongzhimian 2 and Jimian 11 were 47.50 and 32.42, respectively (Table 1). Therefore, FLiC induces systemic disease resistance in upland cotton, and the induction effect in the resistant cultivars is stronger than that in the susceptible cultivars.

**Table 1:** FLiC induces VD disease resistance in 30-day seedlings of different upland cotton varieties.

Treatment	Disease index (DI)	Relative control effect (%)
Jimian 11 (CK)	68.41 ± 0.61a	
Jimian 11 (FLiC)	46.23 ± 1.12c	32.42 ± 1.39b
Zhongzhimian 2 (CK)	55.28 ± 0.61b	
Zhongzhimian 2 (FLiC)	29.00 ± 0.83d	47.50 ± 2.09a

One-way ANOVA (*p* < 0.05) followed by Duncan tests was used for multiple comparisons. The different letters indicate significance at the 0.05 probability level.

### FLiC induces deposition of callose in leaves and lignin in stems

**Figure 3:**
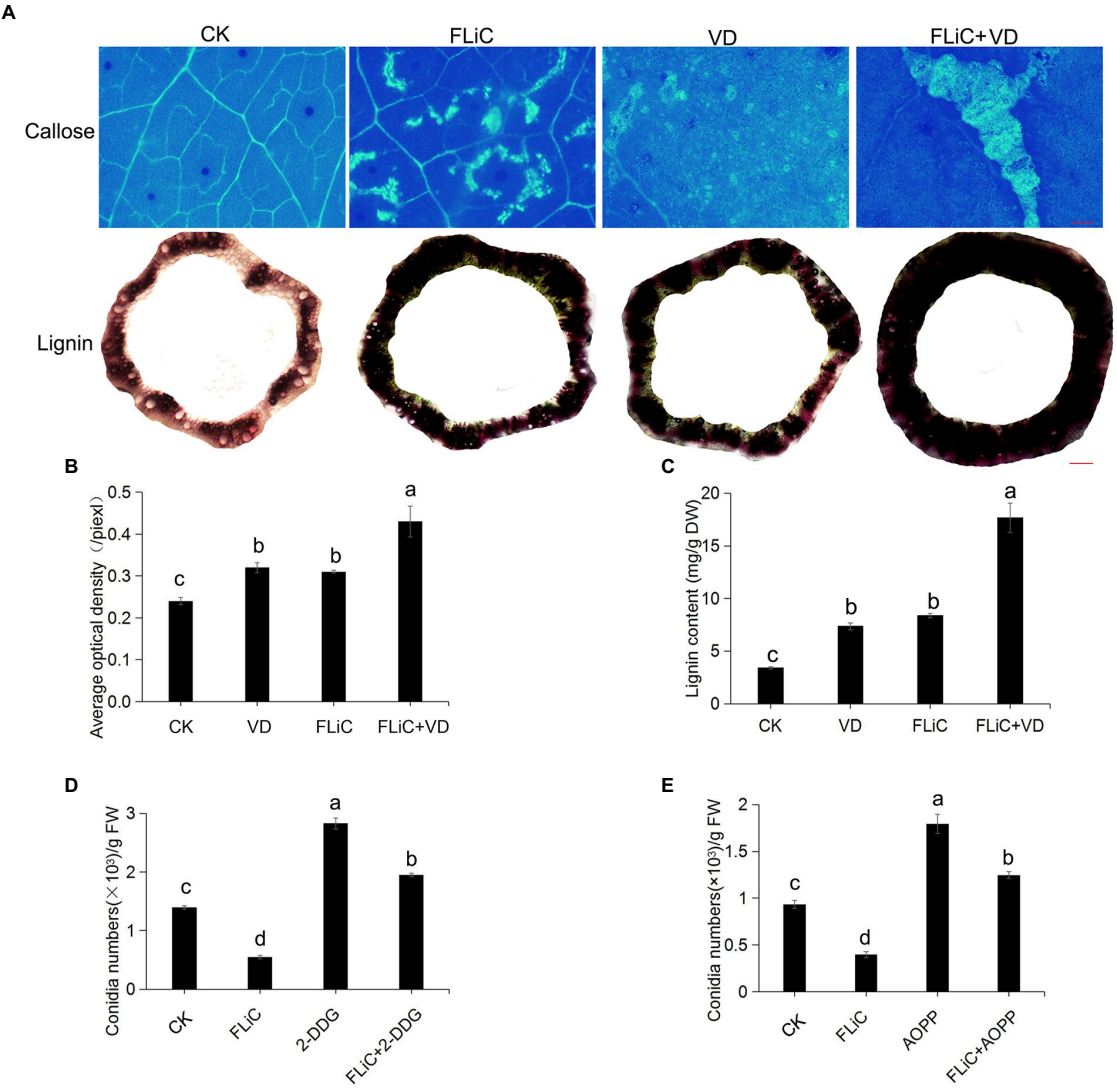
FLiC induces the deposition of lignin and callose in upland cotton. **(A)** FLiC induced the deposition of leaf callose and stem lignin in cotton (callose bars = 1,000 μm; lignin bars = 2,000 μm). The concentration of FLiC was 100 μg/ml, and the cotton leaves were treated for 48 h. **(B)** Statistics of the average optical density of callose. **(C)** Determination of the lignin content. **(D)** Statistics of the bacterial mass after the clearance of callose with 2-DDG. **(E)** Statistics of the bacterial mass after the clearance of lignin with AOPP. The data represent the means ± SDs; *n* = 3. One-way ANOVA (*p* < 0.05) followed by Duncan’s test was used for multiple comparisons. The different letters indicate significance at the 0.05 probability level.

Many studies have identified the accumulation of lignin as a general and basal defense reaction in plant immunity and cotton resistance to VD (Hu et al., 2021). Forty-eight hours after cotton leaves were sprayed with FLiC solution, obvious callose deposits were observed in the leaves of Zhongzhimian 2, and the lignin content in the stems was higher than the control level (Figures 3A–C). To further confirm that the callose and lignin induced by FLiC are involved in enhancing resistance to VD, solutions of 2-deoxy-D-glucose (2-DDG; callose synthesis inhibitor) and α-aminooxyacetic acid-β-phenylpropionic acid (AOPP; Lignin synthesis inhibitor) were sprayed onto cotton leaves, and this spraying decreased the amount of callose and lignin deposition and significantly increased the amount of VD spores on the roots, which decreased the FLiC-induced resistance (Figures 3D,E). Therefore, the results demonstrate that FLiC can induce a strong immune response in cotton and enhance resistance to VD through the production of callose and lignin.

### FLiC-induced resistance depends on changes in defensive enzyme activity

To further verify that FLiC can induce changes in enzyme activity in upland cotton to enhance the defense response, we evaluated the changes in the activities of six enzymes in the leaves of Zhongzhimian 2 sprayed with the FLiC solution only. After the plants were sprayed with FLiC solution, the activities of defense-related enzymes changed to varying degrees (Supplementary Figure S2). Specifically, CAT activity in these plants was significantly higher than that in the control plants on the tenth day, and the activities of PPO, POD, and PAL on the fourth day were significantly higher than those of the control; on the eighth day, the activities were comparable to those of the control; in addition, on the tenth day, all the enzymes except CHI and GLU showed significant increases in activity compared with the activity levels in the control. Moreover, CHI activity showed a significant increase in the treated plants compared with that in the control on the second and fourth days and then began to decrease to the control level. GLU activity was significantly higher on the second day compared with that of the control and then began to decrease. These results showed that the activity of defense-related enzymes exhibits differential changes at different times after inoculation to improve cotton resistance to VD.

### FLiC induces the expression of VD resistance-related genes

Twenty-four hours after the seedlings were sprayed with FLiC solution, defense-related genes such as pathogenesis-related proteins 1 (*PR1*), *CHI*, *GLU*, *POD4*, *ERF5*, *PR10*, lipoxygenase (*LOX*), and *CDN1* were induced to varying degrees (Supplementary Figure S2). Six hours after inoculation, the expression levels of the *MAPK2*, *MAPK6*, and *MAPK7* genes in the roots of seedlings belonging to the treatment group were lower than those in the control roots, and the expression levels of the *MAPK16* gene were higher than the control levels. However, 12 and 24 h after inoculation, the expression levels of the *MAPK2*, *MAPK6*, *MAPK7*, and *MAPK16* genes were basically the same as those in the control plants. These findings indicated that the *MAPK16* gene may be involved in the early immune response induced by FLiC. Six hours after inoculation with VD, the expression levels of the *WRKY5* and *WRKY6* genes were higher than the control levels, whereas those of *WRKY2*, *WRKY3*, and *WRKY*4 were not. At 12 and 24 h after inoculation, the expression levels of *WRKY2*, *WRKY3*, *WRKY4*, *WRKY5*, and *WRKY6* were not significantly different from the control levels. Thus, the *WRKY5* and *WRKY6* transcription factors may be involved in the early immune response induced by FLiC (Supplementary Figure S3).

### FLiC induces an increase in intracellular Ca^2+^

**Figure 4:**
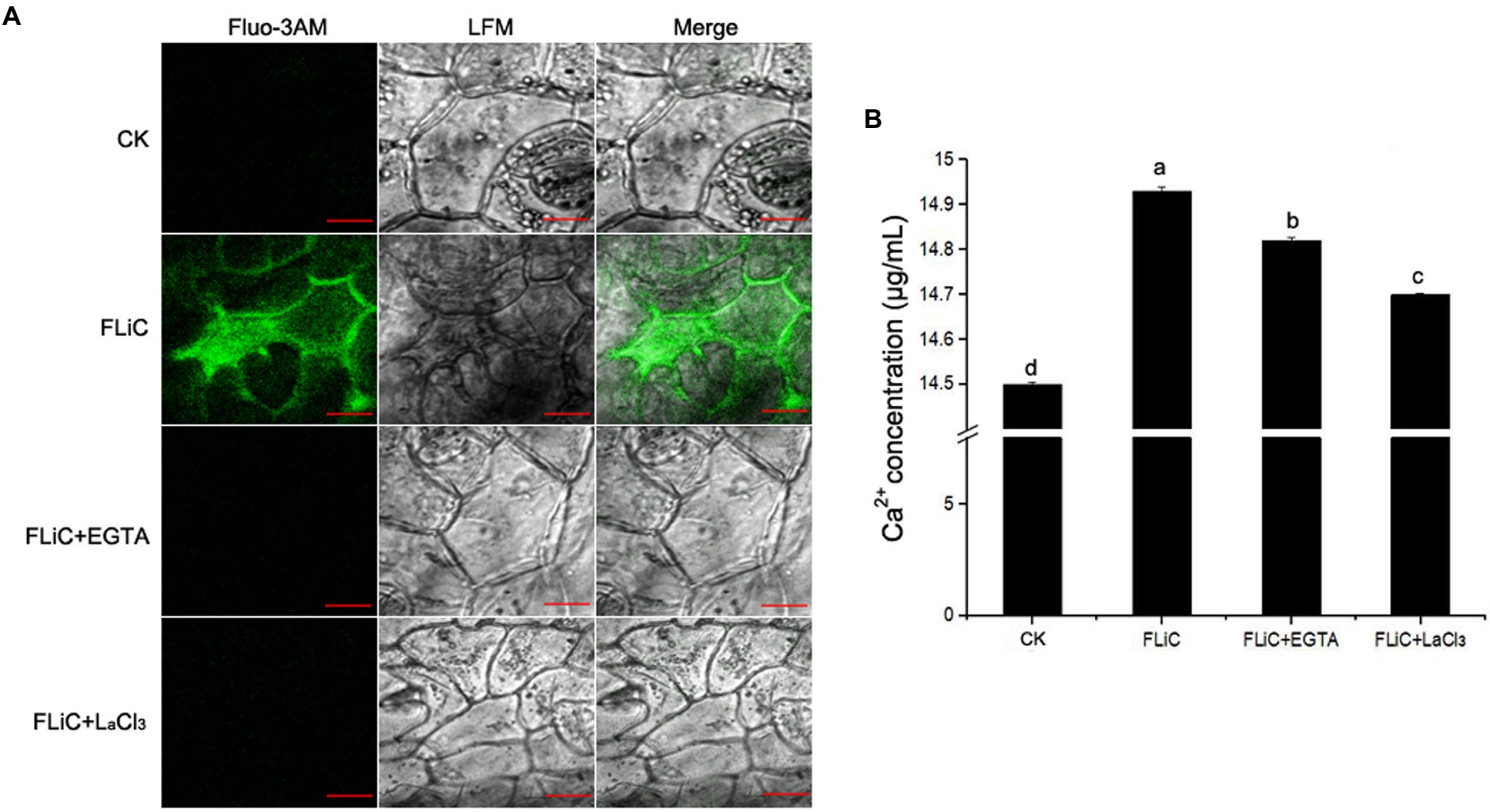
FLiC induces Ca^2+^ production in upland cotton epidermal cells. **(A)** FLiC induces increased intracellular Ca^2+^ content. **(B)** Intracellular Ca^2+^ quantification. The data represent the means ± SDs; *n* = 3. One-way ANOVA (*p* < 0.05) followed by Duncan’s test was used for multiple comparisons. The different letters indicate significant differences at the 0.05 probability level. Bars = 50 μm.

To verify that FLiC protein-induced resistance to VD is related to an increase in intracellular Ca^2+^ content and causes a downstream immune response, epidermal cells were loaded with the Ca^2+^-sensitive fluorescent probe Fluo-3/AM; afterward, solutions of FLiC protein at a concentration of 100 μg/ml were applied, and the effects were observed by LCSM. The results showed that FLiC proteins can cause a significant increase in the fluorescence intensity of epidermal cells (Figure 4). After the seedlings were sprayed with the Ca^2+^ chelating agent ethylene glycol-bis(2-aminoethyl ether)-N,N,N′,N′-tetraacetic acid (EGTA) and a Ca^2+^ channel blocker (LaCl_3_), the fluorescence intensity induced by FLiC was significantly reduced. Therefore, FLiC can induce an increase in the Ca^2+^ concentration in upland cotton epidermal cells.

### The regulatory relationship between Ca^2+^ and H_2_O_2_ was clarified

Twelve hours after cotton leaves were sprayed with FLiC solution, an obvious ROS burst was observed in the leaves (Supplementary Figure S4 A). To determine the relationship between Ca^2+^ and H_2_O_2_, the seedlings were sprayed with CAT (After H_2_O_2_ is synthesized, it will first accumulate outside the cell membrane, and CAT will remove these formed H_2_O_2_ molecules), EGTA (After the Ca^2+^ chelator EGTA enters plant cells, it will chelate Ca^2+^ and reduce the intracellular Ca^2+^ concentration) and LaCl_3_(the Ca^2+^ channel inhibitor LaCl_3_ blocks the influx of extracellular Ca^2+^ into the cell and reduces the intracellular Ca^2+^ concentration) solutions, and this spraying significantly decreased the H_2_O_2_ content in the cotton leaves to the control level. These results indicated that Ca^2+^ acts upstream of H_2_O_2_ and affects the synthesis of H_2_O_2_. Moreover, 5–30 days after the cotton leaves were sprayed with the FLiC solution, the H_2_O_2_ content in the leaves of the seedlings continued to be significantly higher than that of the control. Until the 30th day, the H_2_O_2_ content began to decrease, but the level was still higher than that of the control (Supplementary Figure S4 C). Therefore, FLiC first induces Ca^2+^ increase and then affects the contents of intracellular H_2_O_2_ such that the immune response improves over a long period of time.

### The induction of NO by FLiC improves resistance to VD

Forty-eight hours after inoculation with VD, the number of pathogens colonizing the roots of seedlings sprayed with FLiC solution was significantly lower than that of the control (Supplementary Figure S5 A). To verify that the NO generated after spraying FLiC hinders the colonization of pathogens. The spraying of carboxy-2-phenyl-4,4,5,5-tetramethylimidazoline-3-oxide-1-oxyl (C-PTIO; a scavenger of NO) onto cotton leaves increased the number of colonized pathogens, and the difference between this number and the control was significant (Supplementary Figure S5 D). Five days after the leaves were inoculated with the VD spore solution, lesions were observed on the leaf surface (Supplementary Figure S5 B). The area of damaged leaves in the seedlings sprayed with FLiC solution was significantly lower than that of the control. The spraying of C-PTIO increased the area of damaged leaves in the seedlings of both the FLiC and control groups. These results further indicate that the accumulation of NO induced by FLiC participates in the resistance of upland cotton to VD.

### The relationship among ROS, Ca^2+^, and NO enhance resistance to VD

**Figure 5:**
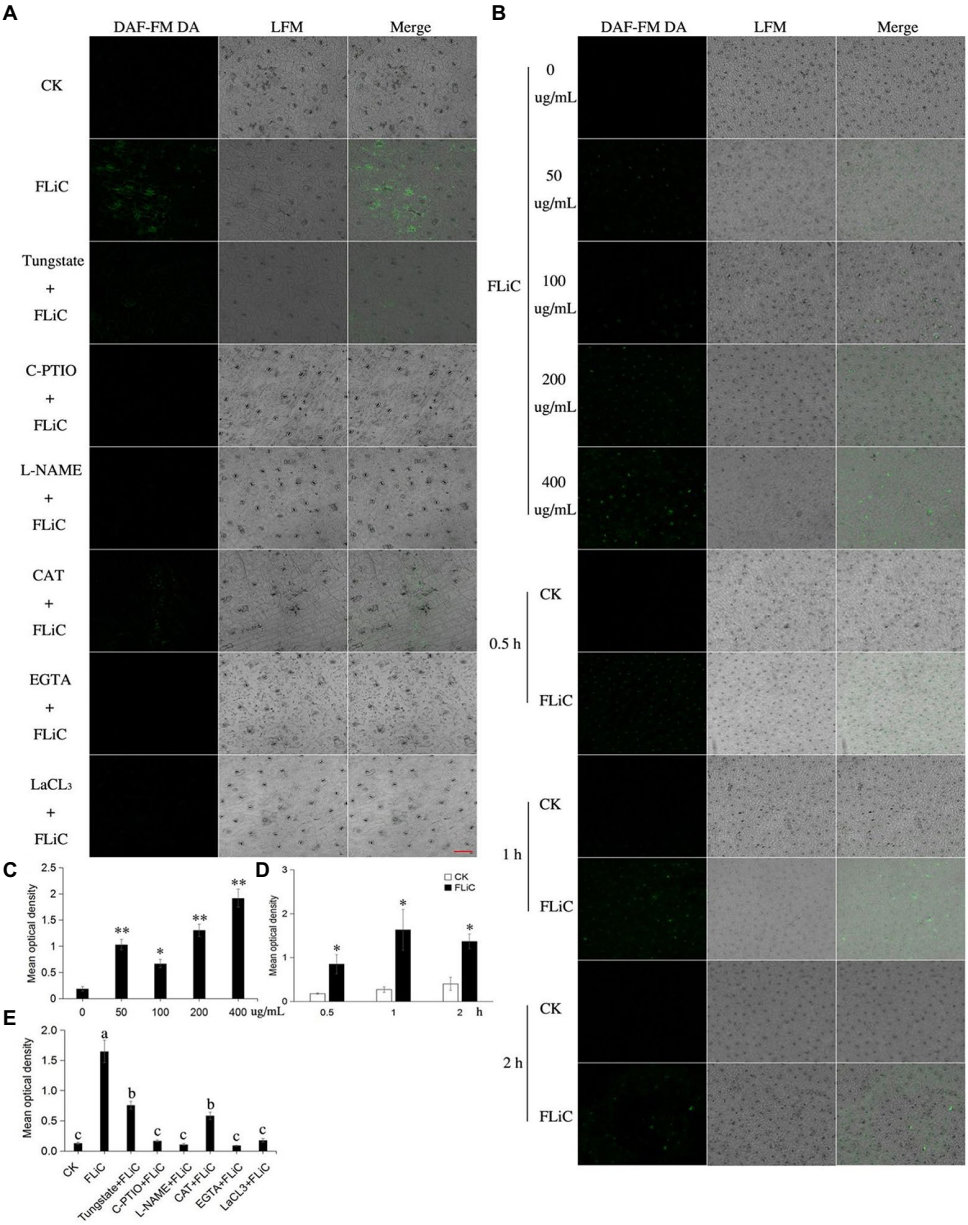
NO production in upland cotton leaves in response to FLiC treatment. **(A)** Fluorescence of upland cotton epidermal cells in response to different treatments (200 μM C-PTIO, 200 μM L-NAME, 100 μM tungstate, 100 unit/ml CAT, 5 mM EGTA, and 200 μM LaCl_3_). **(B)** Fluorescence of upland cotton epidermal cells at different time points after treatment with solutions of different protein concentrations. **(C)** Average fluorescence density of NO in upland cotton epidermal cells treated with solutions of different protein concentrations. **(D)** Average fluorescence density of NO in upland cotton epidermal cells at different time points after treatment. **(E)** Average fluorescence density of NO in upland cotton epidermal cells after different treatments. The data represent the means ± SDs; *n* = 3 (*p < 0.05; **p < 0.01; *t* test). One-way ANOVA (*p* < 0.05) followed by Duncan’s test was used for multiple comparisons. The different letters indicate that the difference between the treatments is significant at the 0.05 probability level. Bars = 20 μm.

To further illuminate the relationship among ROS, Ca^2+^, and NO, strong fluorescence was observed around the epidermal cells and stomata of cotton sprayed with the FLiC solution, which indicated that FLiC can induce the production of NO in cotton epidermal cells of seedlings (Figures 5A,E). The fluorescence intensity of the seedlings was strongest 1 h after being sprayed with 400 μg/ml FLiC solution (Figures 5B,C and D), indicating that the production of NO induced by FLiC depends on the concentration of FLiC and the time after treatment. The spraying of solutions containing the NO scavenger C-PTIO and the NOS pathway inhibitor nitro-L-arginine methyl ester (L-NAME) onto cotton leaves significantly decreased the fluorescence intensity (Figures 5A,E), which indicated that C-PTIO and L-NAME exert an inhibitory effect on FLiC-induced NO production in epidermal cells. The nitrate reductase (NR) pathway inhibitor tungstate had only a weak inhibitory effect on FLiC-induced NO production in epidermal cells. These results indicated that FLiC induces the production of NO in epidermal cells; NO is synthesized mainly through the nitric oxide synthase (NOS) pathway. The spraying of a solution containing CAT, EGTA, and LaCl_3_ onto cotton leaves significantly decreased the NO content. These results showed that Ca^2+^ and H_2_O_2_ are involved in the production of NO. After the seedlings were sprayed with C-PTIO and L-NAME solutions, FLiC could still induce increased H_2_O_2_ production. These results showed that the production of H_2_O_2_ occurs independently of the NO content. However, excessive amounts of NO exert an inhibitory effect on FLiC-induced H_2_O_2_ production (Supplementary Figure S4 B). The spraying of the seedlings with EGTA and LaCl_3_ solutions significantly decreased the content of H_2_O_2_ (Supplementary Figures S4A,B). Therefore, in the immune response induced by FLiC, Ca^2+^ induces H_2_O_2_ and participates in the production of H_2_O_2_; H_2_O_2_ subsequently stimulates the production of NO; and excessive NO inhibits the production of H_2_O_2_.

### Significant metabolic pathways enriched in transcriptome results

To analyze which metabolic pathways were associated with the transcriptome results, we performed GO and KEGG enrichment analyses. The three most significant GO enrichment pathways were metal ion transmembrane transporter activity, cellular potassium ion transport, and potassium ion transmembrane transport. The differentially expressed genes were enriched in the following pathways (in descending order): oxidoreductase activity (22 DEGs), metal ion transport (seven DEGs), metal ion transmembrane transporter activity (seven DEGs), and monovalent inorganic cation transmembrane transporter activity (six DEGs). In addition, significantly enriched to calcium antiporter activity. The three most significant pathways identified in the KEGG enrichment analysis were glycolysis/gluconeogenesis, carbon fixation in photosynthetic organisms, and diterpenoid biosynthesis. The genes were enriched in the following pathways (in descending order): glycolysis/gluconeogenesis (five DEGs), phenylpropanoid biosynthesis (four DEGs), and carbon fixation in photosynthetic organisms (three DEGs). These results suggest that the resistance induced by FLiC in cotton is related to calcium transporter activity.

### The cation/proton exchangers gene family negatively regulates cotton plant resistance to VD

**Figure 6:**
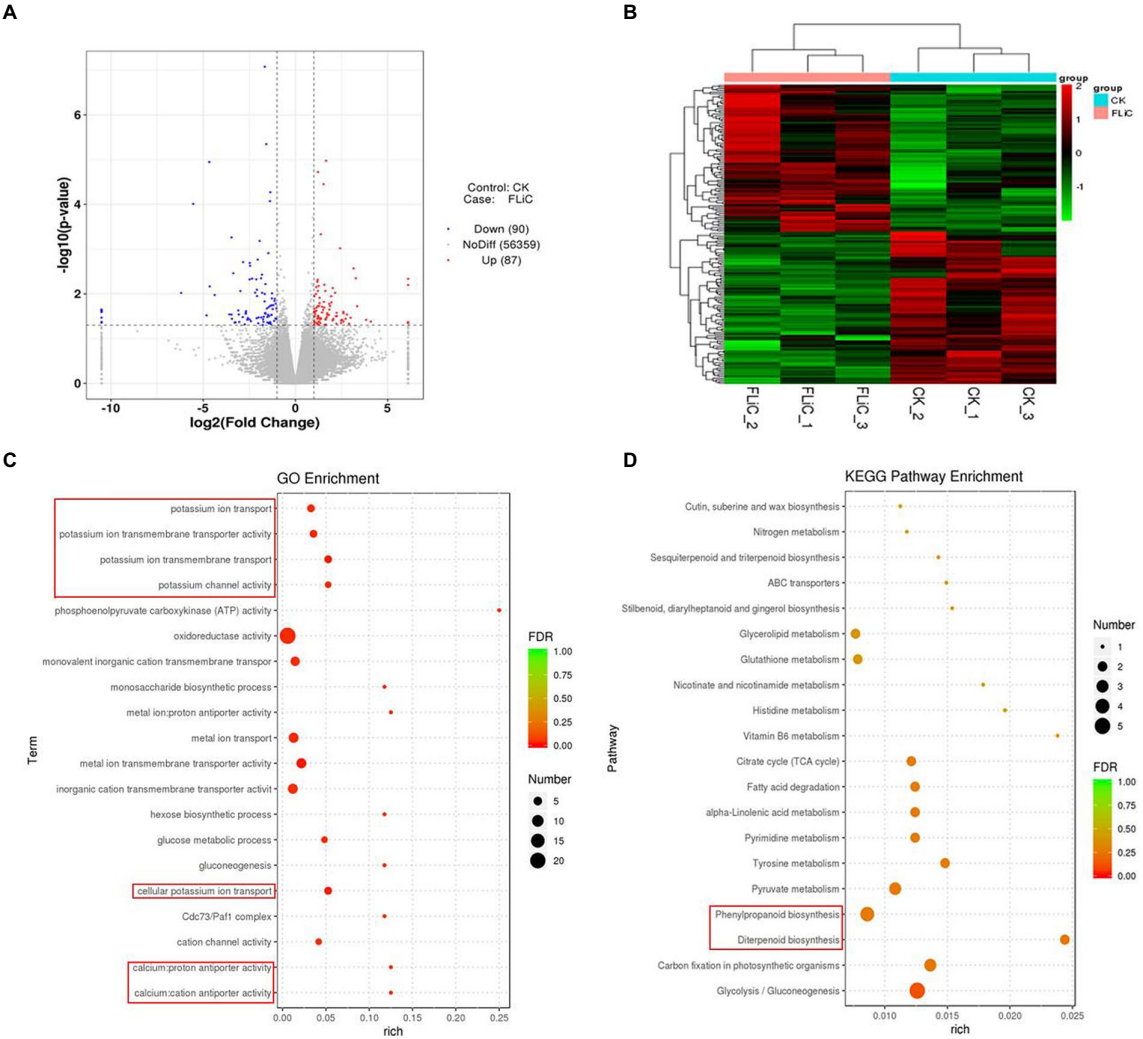
Pathways in which the DEGs are significantly enriched according to the transcriptomic data. **(A)** Volcano plot of DEGs. **(B)** DEG cluster analysis. **(C)** Bubble chart of the top 20 metabolic pathways enriched in GO terms. **(D)** Bubble chart of the top 20 metabolic pathways enriched in KEGG pathways.

The above-described studies confirmed that FLiC induces the immune response in cotton due to increased intracellular Ca^2+^ content, and the coordinated regulation of Ca^2+^, H_2_O_2_, and NO enhances the resistance to VD. To clarify which metabolic pathways regulate intracellular Ca^2+^ content, we analyzed the transcriptome results. The RNA-seq results showed that 87 genes were upregulated and 90 genes were downregulated. Twelve genes were randomly selected from the RNA-seq results to verify the transcriptome results by qRT–PCR. The results showed that the expression trends of 11 of the 12 genes were the same as those identified from the transcriptome data, with an accuracy rate of 91.67%. This finding shows that the transcriptome results are credible (Supplementary Figure S6). The significantly enriched DEGs were classified and clustered based on the results (Figures 6A,B). The DEGs were significantly enriched in related disease-resistant metabolic pathways, such as those involving calcium antiporter activity, potassium ions, diterpenoid biosynthesis, and phenylpropanoid biosynthesis (Figures 6C,D). Further analysis revealed that calcium antiporter activity is regulated by the cation/proton exchangers gene family. The expression level of this gene family in the plants sprayed with FLiC solution was lower than the control level. Therefore, this gene family may negatively regulate cotton plant resistance to VD.

### The immune response is enhanced after *GhCAX3* silencing

**Figure 7:**
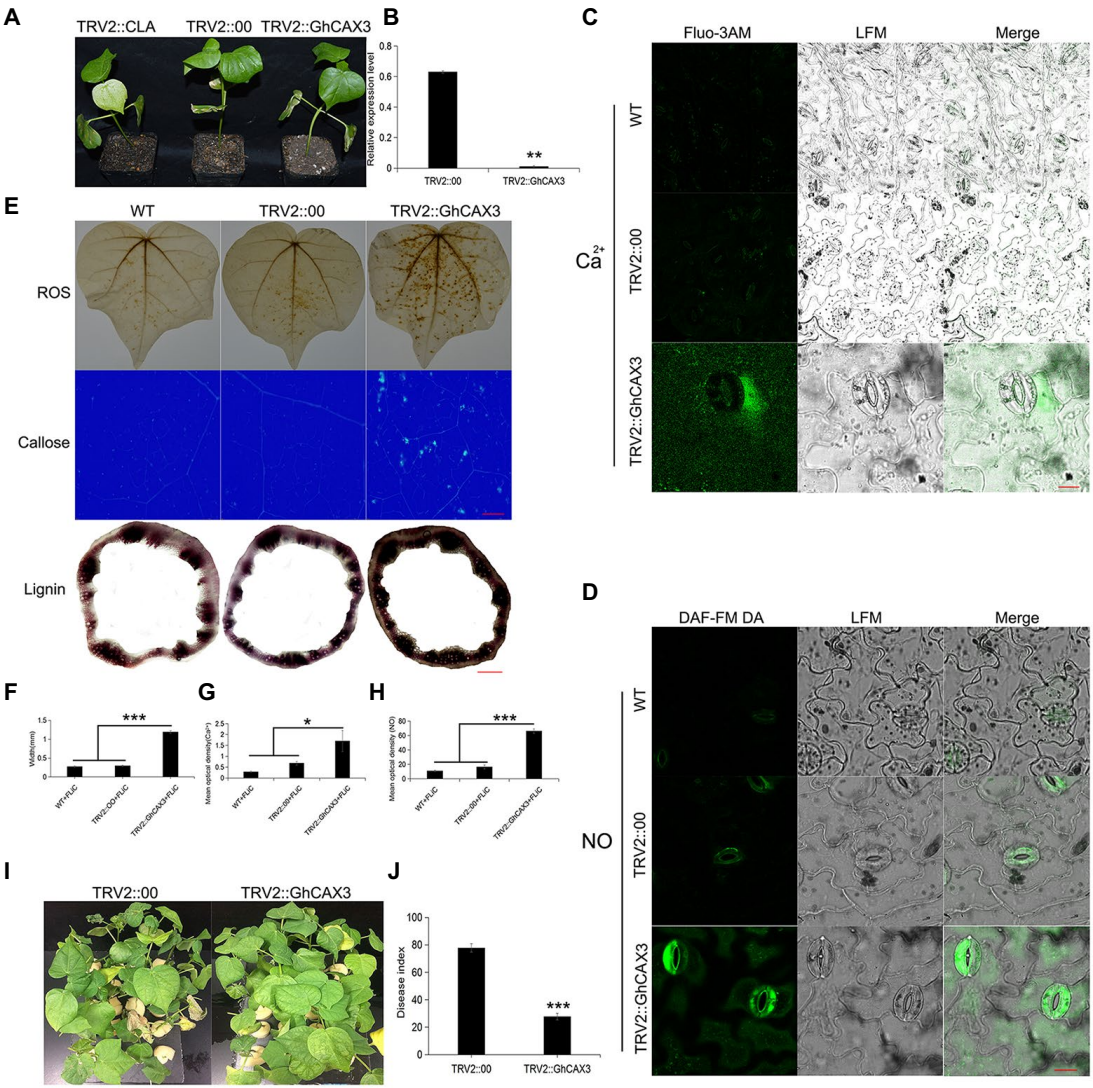
Anti-disease substances increase after the silencing of *GhCAX3*. **(A)** Imaging of plants transformed with TRV2::GhCAX3 at 10 days after gene silencing; TRV2::CLA served as the positive control, and TRV2::00 was the negative control. **(B)** *GhCAX3* gene silencing efficiency. **(C)** Intracellular Ca^2+^ content of cotton after *GhCAX3* gene silencing. **(D)** Intracellular NO content of cotton after *GhCAX3* gene silencing. **(E)** ROS, callose, and lignin contents after *GhCAX3* gene silencing. **(F)** Determination of the lignin content. **(G)** Quantification of intracellular Ca^2+^ in cotton after *GhCAX3* gene silencing. **(H)** Intracellular NO quantification in cotton after *GhCAX3* gene silencing. **(I)** Detection of disease resistance after *GhCAX3* gene silencing. **(J)** Investigation of disease index after *GhCAX3* gene silencing. The data represent the means ± SDs; *n* = 3 (*p < 0.05; **p < 0.01; ***p < 0.001; *t* test). Bars **(C,D)** = 50 μm. Bars **(E)** = 2000 μm.

Pathogen induced Ca^2+^ signaling and homeostasis is mediated by two factors, the Ca^2+^ influx channels and the Ca^2+^ efflux pumps/exchangers (Ca^2+^-ATPase or CAX type Ca^2+^/H^+^ exchangers). To verify that the cation/proton exchanger 3 (*GhCAX3*; CAX type Ca^2+^/H^+^ exchangers) gene negatively regulates cotton resistance to VD, the *GhCAX3* gene was silenced. After true leaves of the positive control became albino, the silencing efficiency of *GhCAX3* was determined to have reached 62% (Figures 7A,B). Twenty-five days after inoculation with VD, the disease index was determined, and the TRV2::GhCAX3 group exhibited a significant lower index than the control TRV2::00 group (Figures 7I,J). After the *GhCAX3* gene was silenced, the intracellular Ca^2+^ and NO contents in the epidermis of leaves of plants sprayed with FLiC solution were significantly higher than those of wild-type (WT) plants and plants transformed with an empty vector (TRV2::00; Figures 7C,D). The levels of ROS, callose and lignin om the sprayed plants were significantly higher than those in WT and TRV2::00-transformed plants (Figures 7E–H). Obviously, silencing of *GhCAX3* through VIGS decreases the Ca^2+^ extrusion from the cytosol and thus causing the enhanced Ca^2+^ concentration as well as Ca^2+^-dependent ROS and NO signaling. Therefore, FLiC negatively regulates the expression of the *GhCAX3* gene to enhance the resistance of seedlings to VD.

### Transgenic Arabidopsis overexpressing the *FLiC* gene exhibits increased resistance to VD and increased expression of VD resistance genes

Arabidopsis plants overexpressing the *FLiC* gene exhibited enhanced disease resistance to VD compared with WT Arabidopsis plants (Supplementary Figure S7 A). The expression of the *FLiC* gene was significantly increased 24 h after inoculation (Supplementary Figure S7 B). Therefore, overexpression of the *FLiC* gene in Arabidopsis might improve resistance to VD. To verify that Arabidopsis plants overexpressing the *FLiC* gene exhibit enhanced resistance to VD, the expression levels of LOX, PAL, PR1, and vegetative storage protein (VSP) genes were measured, and the results showed that the expression of the four disease resistance genes were significantly increased. Therefore, overexpression of the *FLiC* gene in Arabidopsis can effectively increase the expression of disease resistance genes related to the salicylic acid (SA) and jasmonic acid (JA) signaling pathways and enhance resistance to VD. The expression level of the glutathione peroxidase (*GPX7*) gene was similar to or lower than that of the control; *POD* expression more obvious in the *FLiC*-overexpressing plants exposed to VD than in the control plants exposed to VD; and the glutathione S-transferase (*GSTU3*) gene showed significantly increased expression (Supplementary Figure S7). Therefore, in transformed Arabidopsis plants, the *FLiC* gene can effectively upregulate the expression of ROS and NO signal-related genes in roots and can enhance resistance to VD.

## Discussion

### Ca^2+^, NO, and H_2_O_2_ synergistically enhance upland cotton resistance to VD

**Figure 8:**
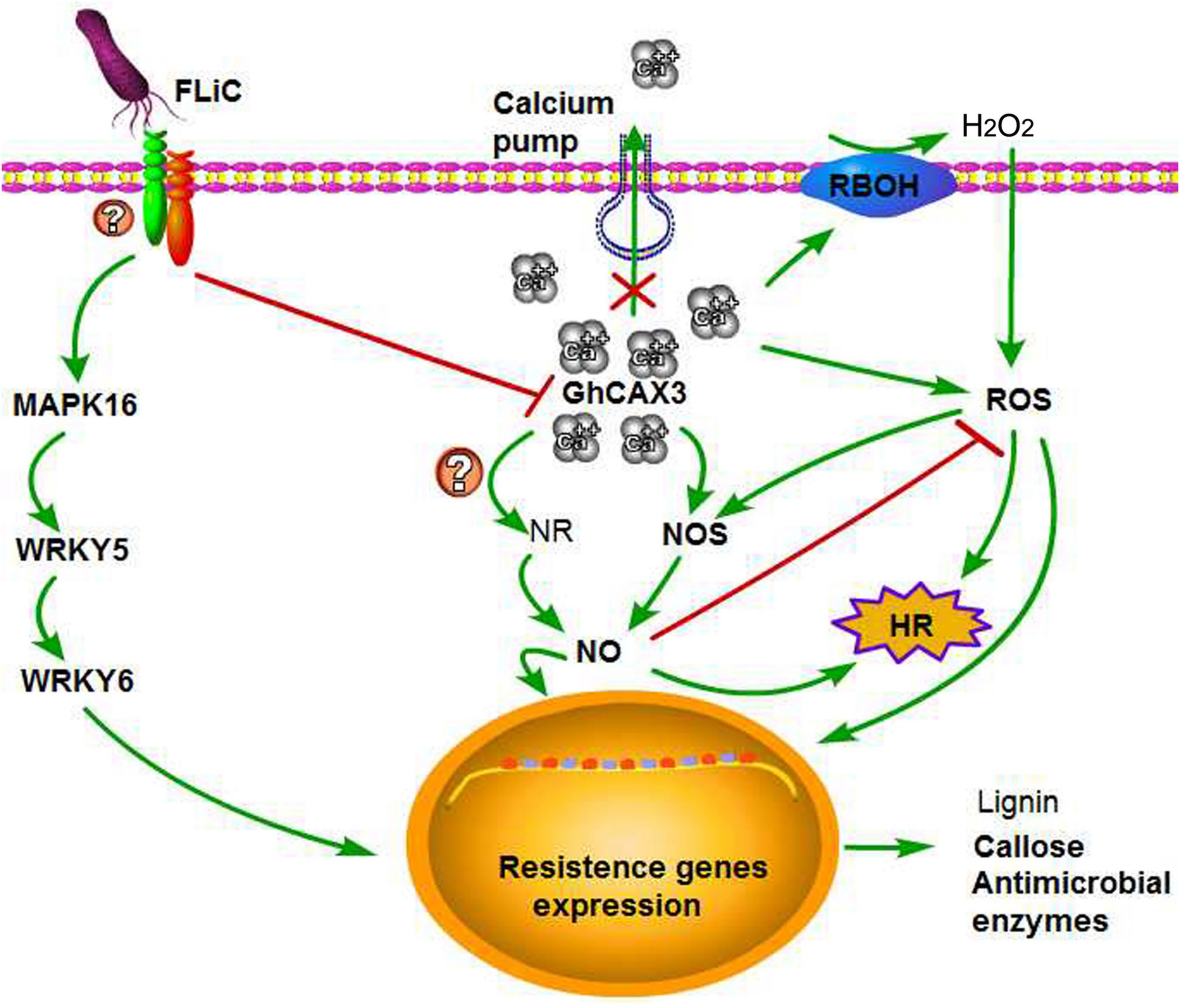
Possible explanation of the FLiC-induced resistance signaling pathway. FLiC increases the intracellular calcium ion content by inhibiting the expression of the calcium ion pump-regulating gene *GhCAX3*, which in turn induces increases in the H_2_O_2_ and NO contents and thereby improves the resistance of cotton to Verticillium wilt.

The relationships among Ca^2+^, NO, and H_2_O_2_ in plants and the regulatory mechanisms through which plant disease resistance can improve remain unknown. We found that NO participates in the signal transduction of plants in response to various biotic and abiotic stresses (Yan et al., 2007; Sang et al., 2008; Martínez-Medina et al., 2019). Induction with the elicitor Ca^2+^ increases the production of NO in tobacco and grape, and NO can in turn cause an increase in the intracellular Ca^2+^ concentrations (Lamotte et al., 2004; Vandelle et al., 2006; Besson-Bard et al., 2008). These investigations indicate an interaction between Ca^2+^ and NO signals. An increase in the Ca^2+^ concentration in mesophyll cells was found to be needed for the cells to produce ROS after pathogen infection (Qiao et al., 2015). Exogenous H_2_O_2_ application to *Arabidopsis thaliana* root epidermis results in dose-dependent transient increases in the net Ca^2+^ influx (Demidchik et al., 2007). These results indicate an interaction between Ca^2+^ and H_2_O_2_ signals. The synergistic effect of NO and H_2_O_2_ triggers the death of hypersensitive cells, but the absence of either one of these factors prevents the induction of cell death (Tada et al., 2004). By affecting the activity of NO synthase, H_2_O_2_ can induce the synthesis and accumulation of NO (Zhang et al., 2007). Similarly, NO can regulate the H_2_O_2_ levels, and H_2_O_2_ is involved in mediating the NO-induced resistance of tomato to *Rhizopus nigricans* (Fan et al., 2008). These results showed that transient changes in the contents of NO and H_2_O_2_ can activate a series of physiological responses in plants and that these compounds can interact to regulate the same or related signaling pathways to enhance a certain response. Ca^2+^ and H_2_O_2_ act upstream of NO production to induce the death of hypersensitive cells (Qiao et al., 2015). Our results showed that Ca^2+^ and H_2_O_2_ also act upstream of NO. However, whether the content of NO negatively alters the content of H_2_O_2_ is unclear. Our results suggest that FLiC binds to membrane receptors and negatively regulates *GhCAX3* to increase the intracellular content of Ca^2+^ and induce the production of H_2_O_2_ and NO; that H_2_O_2_ induces the production of NO, whereas NO inhibits the synthesis of H_2_O_2_; and that H_2_O_2_ and NO can induce the production of defense response-related compounds. These findings show that Ca^2+^, NO, and H_2_O_2_ synergistically regulate the resistance of upland cotton plants to VD, which is induced by FLiC (Figure 8). Our results provide the first demonstration that FLiC negatively regulates *GhCAX3* to enhance the resistance of upland cotton to VD.

### Metabolic pathways through which FLiC protein induces an immune response

The mechanisms through which full-length FLiC protein affects the plant immune response have not yet been reported. We cloned a newly identified flagellin gene, *FLiC*, which encodes the same amino acid sequence as Flg22. In cotton, FLiC can induce immune responses, such as *MAPK* cascade reactions, and upregulation of key genes involved in disease resistance pathways, such as those involving SA, JA, and ETH (Supplementary Figure S4). According to a transcriptome analysis, the DEGs identified after application of the protein to cotton (by spraying of solutions) were significantly enriched in pathways involving potassium ions, Ca^2+^, diterpenoid synthesis, phenylpropanoid biosynthesis, lignin biosynthesis, nitrogen metabolism, and other disease resistance-related metabolic pathways. Therefore, the disease resistance induced by FLiC protein in cotton is related to the activation of these pathways. Moreover, the *CAX3* gene has not been found to regulate the Ca^2+^ levels or participate in the disease resistance of cotton. In this study, we found that *GhCAX3* negatively regulated the Ca^2+^ levels and thus hampered the VD resistance of cotton plants sprayed with FLiC solution. Plant Ca^2+^ signals are involved in a wide array of intracellular signaling pathways following pathogen invasion (Kanchiswamy et al., 2013; Aldon et al., 2018; Wang et al., 2019). Ca^2+^-dependent protein kinases have been predicted to mediate signaling following Ca^2+^ influx after pathogen infection (Kanchiswamy et al., 2013; Ma et al., 2013; Bredow and Monaghan, 2019). The pathogen-associated molecular patterns involving bacterial flagellin are the most intensively studied PAMPs (Wang, 2012). After the treatment of *Arabidopsis thaliana* with Flg22, Ca^2+^ channels and their involvement in the activation of the stomatal closure mechanism in the process of immune signal transduction indicated the specificity of the Ca^2+^ influx mechanism in response to different stresses (Thor et al., 2020). Moreover, after treatment with Flg22, *Arabidopsis thaliana* double-knockout plants (in which the genes encoding the tonoplast-targeted pump aca 4/11 were knocked out) showed higher basal Ca^2+^ levels and greater Ca^2+^ signals than WT plants (Hilleary et al., 2020). The HopZ-activated resistance 1 resistosome acts as a calcium-permeable cation channel to trigger plant immunity and cell death (Bi et al., 2021). Taken together, these findings show that calcium transporters can negatively regulate intracellular Ca^2+^content. However, the *GhCAX3* gene has not been previously found to regulate the Ca^2+^ levels or participate in the disease resistance of cotton. Through transcriptome analysis, we found that *GhCAX3* negatively enhanced upland cotton resistance to VD. Many studies have investigated the ability of flagellin to induce plant immunity, but whether flagellin regulates *CAX3* to induce plant immunity has not been reported. We cloned a new type of flagellin gene, *FLiC*, which has the same encoded amino acid sequence as that of Flg22. Whether Flg22 regulates *CAX3* to induce plant immunity has not been reported, and how Flg22 regulates the relationship among Ca^2+^, NO, and H_2_O_2_ to improve disease resistance has not yet been elucidated. We found that the silencing of *GhCAX3* in cotton increased the intracellular Ca^2+^ content and thereby induced increases in the contents of H_2_O_2_, NO, and disease resistance-related substances after the plants were sprayed with FLiC solution. These results showed that FLiC inhibits the expression of *GhCAX3*, which increases the intracellular Ca^2+^ content and induces increases in the H_2_O_2_ and NO contents to enhance upland cotton resistance to VD.

### *FLiC*-transformed Arabidopsis exhibits increased resistance to VD

The flagellin gene of *Bacillus subtilis* has been successfully transferred into rice, which results in increased resistance to rice blast and bacterial streaks (Wang et al., 2014, 2015). The inoculation of Arabidopsis plants overexpressing *FLiC* with VD significantly increased the relative expression levels of the SA and JA defense signal-related genes *LOX*, *PAL*, *PR1*, and *VSP*, and the expression levels of both *LOX* and *PR1* increased 40-fold. Moreover, the expression of the *GSTU3*, *GPX7*, and *POD* genes, which are related to the ROS and NO signaling pathways, changed to varying degrees. Therefore, the *FLiC* transgene in Arabidopsis can induce the expression of key genes involved in the SA, JA, ROS, and NO signaling pathways to improve plant disease resistance. Therefore, our research not only revealed a possible use of a new full-length flagellin gene but also used upland cotton as a research material to explore a new approach for inducing an immune response in plants and provide a new protein that can improve the resistance of upland cotton to VD.

## Conclusion

Flagellin C induced HRs in tobacco and immune responses in cotton. FLiC reduces the expression of *GhCAX3* to increase the intracellular Ca^2+^ content and then stimulate the increase of intracellular H_2_O_2_ and NO content. The coordinated regulation of Ca^2+^, H_2_O_2_, and NO enhanced the resistance to VD, and transgenic Arabidopsis plants overexpressing *FLiC* exhibited significant resistance to VD.

## Data availability statement

The original contributions presented in the study are publicly available. This data can be found at: NCBI, PRJNA847674.

## Author contributions

CT: conceptualization, supervision, and funding acquisition. HZ, YW, and YZ: data curation. HZ and YW: investigation. HZ and HN: formal analysis and writing–original draft. HZ, YX, and CT: writing–review and editing. All authors contributed to the article and approved the submitted version.

## Funding

This work was financially supported by the National Key R&D Program of China (2016YFD0102105) and the Postgraduate Research & Practice Innovation Program of Jiangsu Province (KYCX20_0582).

## Conflict of interest

The authors declare that the research was conducted in the absence of any commercial or financial relationships that could be construed as a potential conflict of interest.

## Publisher’s note

All claims expressed in this article are solely those of the authors and do not necessarily represent those of their affiliated organizations, or those of the publisher, the editors and the reviewers. Any product that may be evaluated in this article, or claim that may be made by its manufacturer, is not guaranteed or endorsed by the publisher.

## Figures and Tables

**Table:** 

2-DDG	2-deoxy-D-glucose
AOPP	α-aminooxyacetic acid-β-phenylpropionic acid
Ca^2+^	Calcium ion
CAT	Catalase
CHI	Chitinase
C-PTIO	Carboxy-2-phenyl-4,4,5,5-tetramethylimidazoline-3-oxide-1-oxyl
DAB	Diaminobenzidine
DEG	Differentially expressed gene
EGTA	Ethylene glycol-bis(2-aminoethylether)-N,N,N′,N′-tetraacetic acid
ETI	Effector-triggered immunity
FLiC	Flagellin C
*GhCAX3*	*Gossypium hirsutum* cation/proton exchanger 3
GLU	Glucanase
GO	Gene ontology
*GPX7*	Glutathione peroxidase
*GSTU3*	Glutathione S-transferase
H_2_O_2_	Hydrogen peroxide
IPTG	Isopropyl-β-D thiogalactopyranoside
JA	Jasmonic acid
KEGG	Kyoto encylopedia of genes and genomes
L-NAME	Nitro-L-arginine methyl ester
*LOX*	Lipoxygenase
NADPH	Nicotinamide adenine dinucleotide phosphate
NO	Nitric oxide
PAL	Phenylalanine ammonia lyase
POD	Peroxidase
PPO	Polyphenol oxidase
*PR1*	Pathogenesis-related protein 1
PTI	Pattern-triggered immunity
qRT–PCR	Quantitative reverse transcription PCR
ROS	Reactive oxygen species
SA	Salicylic acid
VD	*Verticillium dahliae*
*VSP*	Vegetative storage protein
