# Supplementary material for: Flagellin C decreases the expression of the Gossypium hirsutum cation/proton exchanger 3 gene to promote calcium ion, hydrogen peroxide, and nitric oxide and synergistically regulate the resistance of cotton to Verticillium wilt
Source: Front Plant Sci. 2022 Sep 21;13:969506. doi: 10.3389/fpls.2022.969506 (PMC9532700; doi:10.3389/fpls.2022.969506)
Supplement: Supplementary file 1 [file Data_Sheet_1.docx]

Supplementary Material

## Supplementary Figures


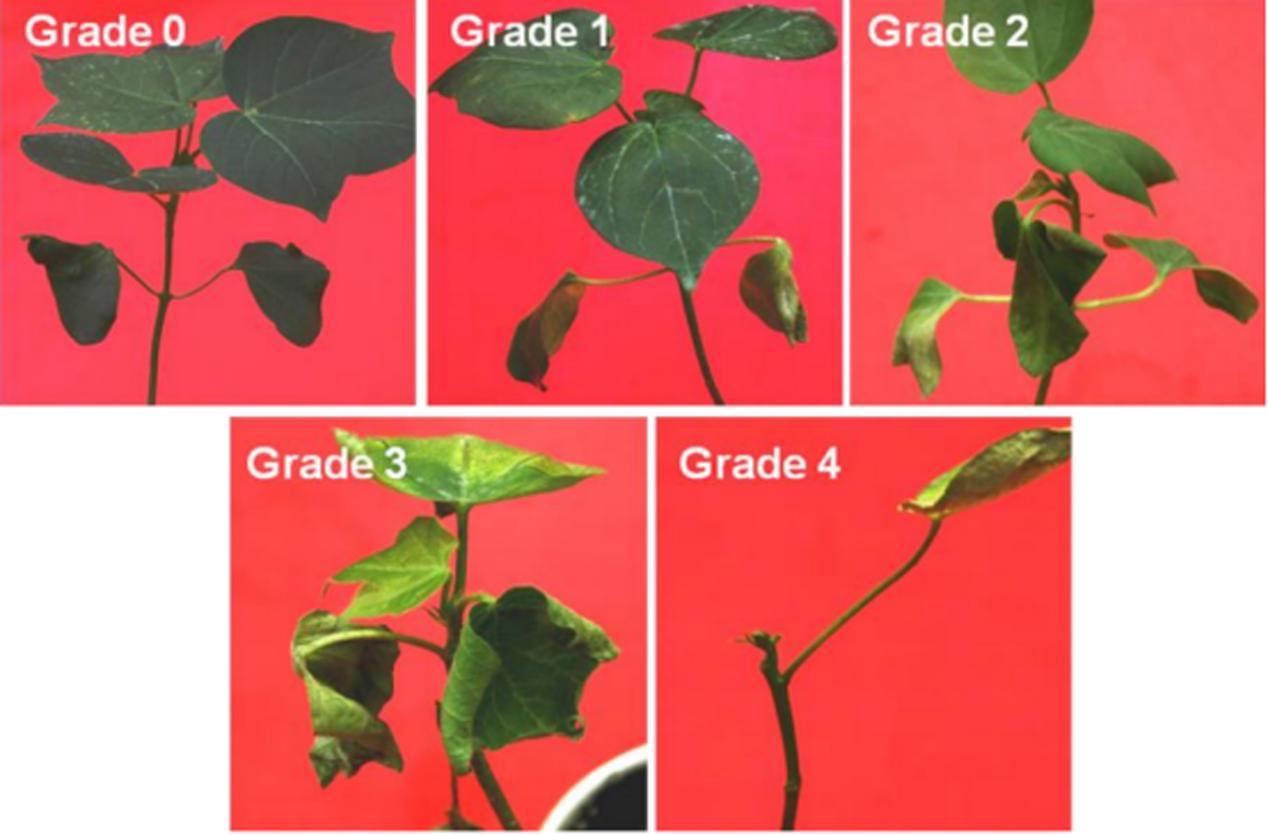


**Supplementary Figure 1** Verticillium wilt disease grade identification of cotton (Liu et al., 2014). Grade 0, healthy with no symptoms on the leaves; Grade1, one or two cotyledons showing symptoms; Grade 2, a single true leaf showing symptoms; Grade 3, more than two leaves showing symptoms; Grade 4,  plant death.


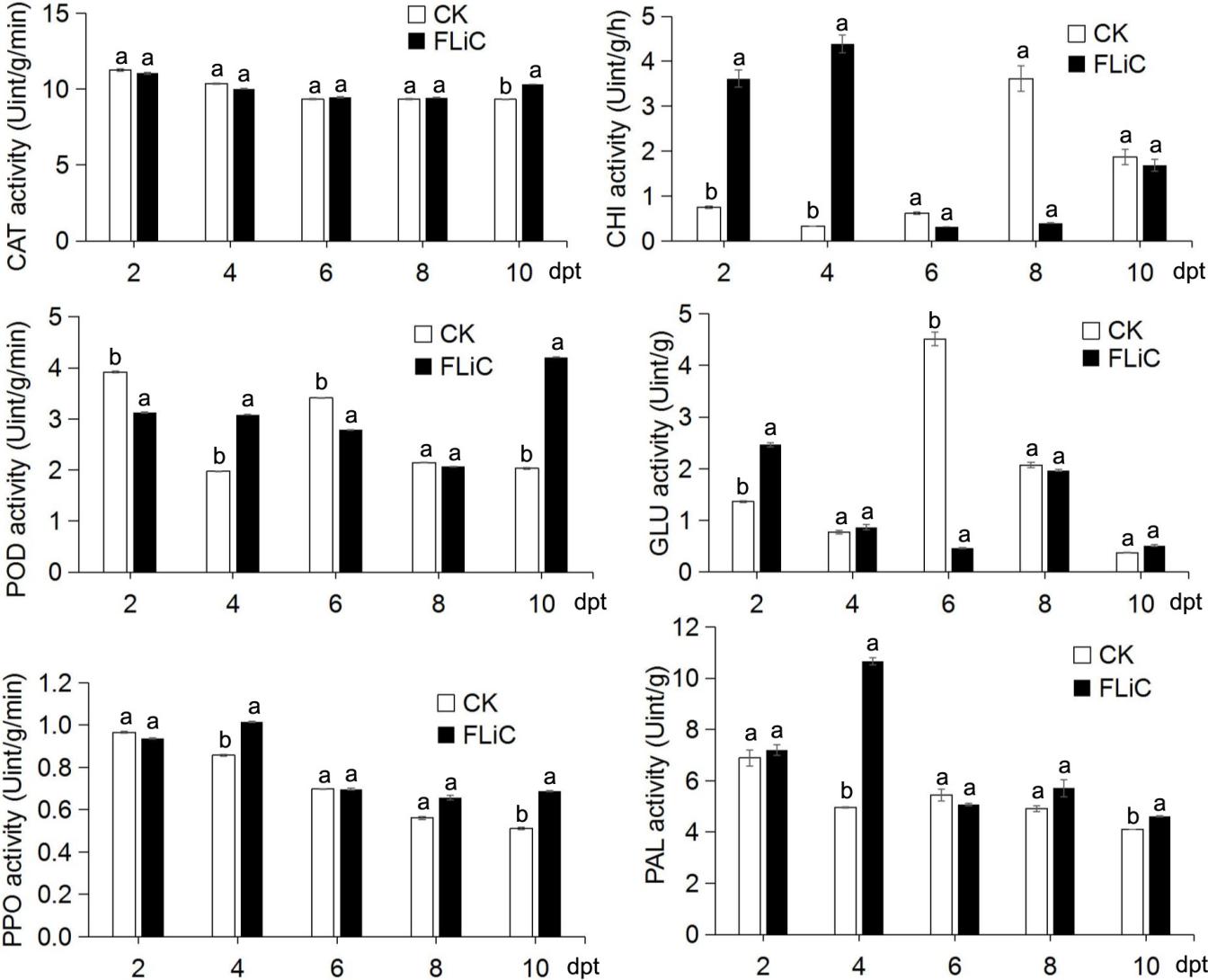


**Supplementary Figure 2** FLiC induces changes in defense-related enzyme activities. The data represent the means ± SDs; n = 3. The different letters indicate statistically significant differences (p < 0.05), as determined by t tests.


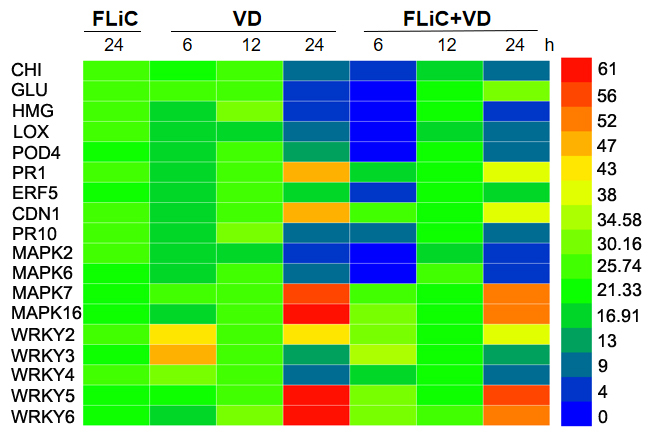


**Supplementary Figure 3** Flagellin FLiC induces defense-related gene expression


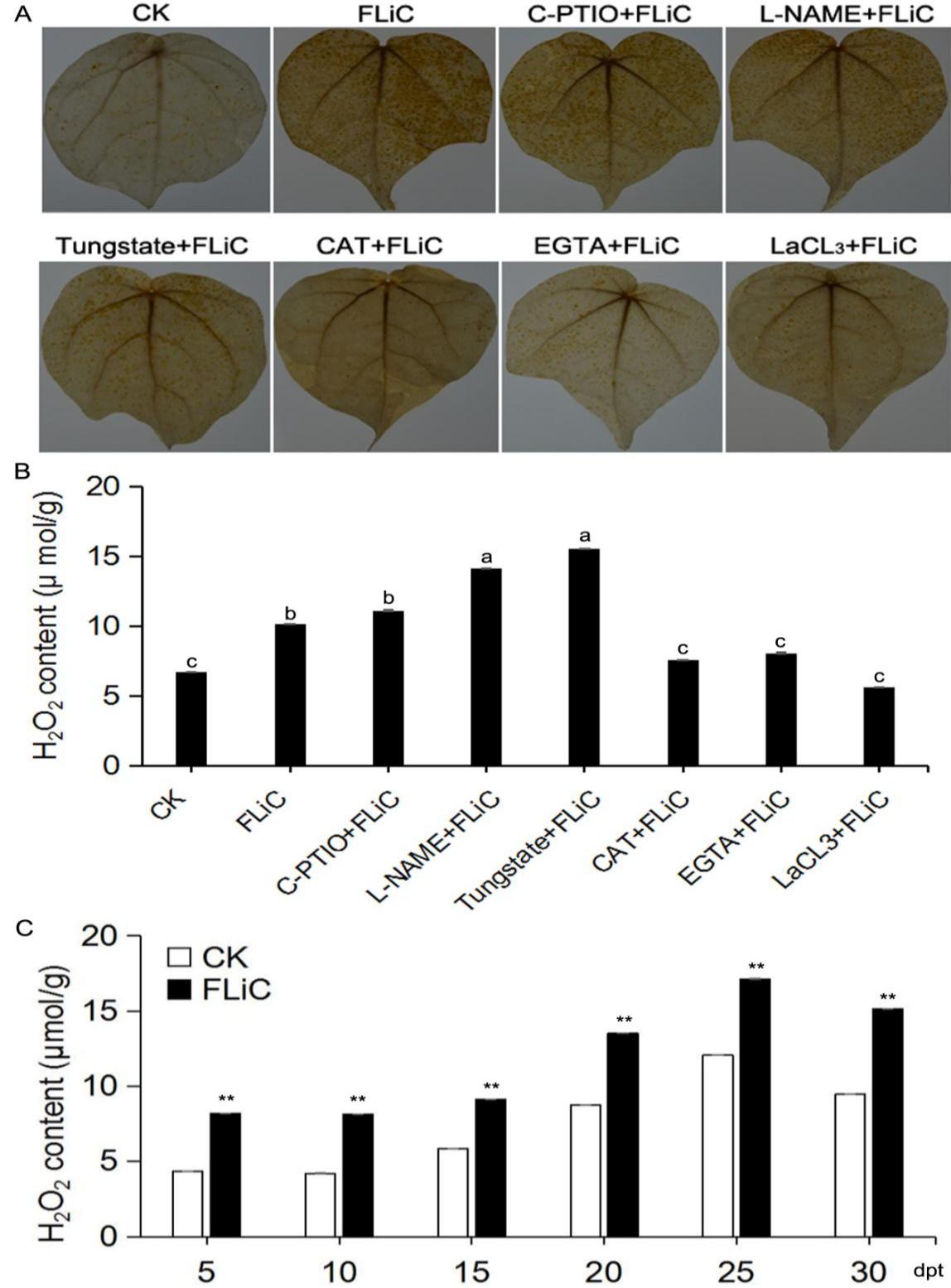


**Supplementary Figure 4** Determination of H_2_O_2_ contents in cotton treated with different chemicals. (A) H_2_O_2_ accumulation in cotton leaves after different chemical treatments. The effects of the NO scavenger C-PTIO, NO synthesis inhibitors L-NAME and sodium tungstate, H_2_O_2_ scavenger CAT, Ca^2+^-chelating agent EGTA, and Ca^2+^ channel inhibitor LaCl_3_ on FLiC-induced H_2_O_2_ production are shown. The brown substance indicates H_2_O_2_ in the leaves. (B) H_2_O_2_ contents in cotton leaves treated with different chemicals. (C) Changes in H_2_O_2_ content within one month after FLiC spraying on cotton leaves. The data represent the means ± SDs; n = 3 (**, p < 0.01; t test). One-way ANOVA (P< 0.05) followed by Duncan’s test was performed for multiple comparisons. The different letters indicate significant differences at the 0.05 probability level.


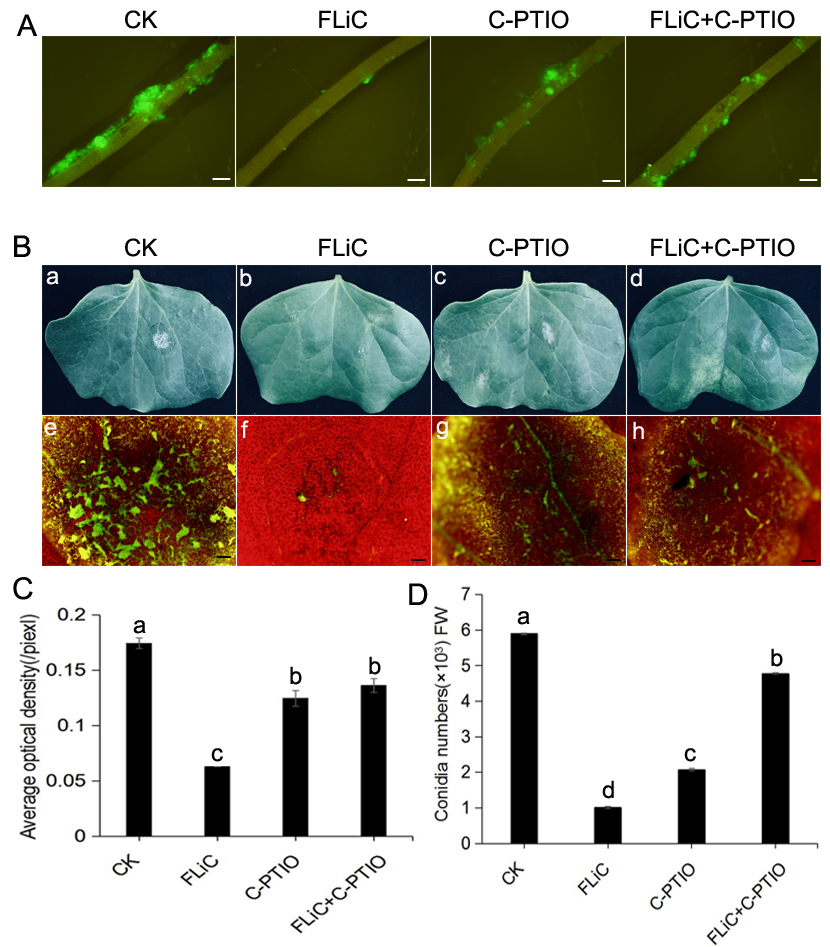


**Supplementary Figure 5** NO is involved in FLiC-induced disease resistance. **(A)** Colonization of fluorescently labeled VD on upland cotton roots. **(B)** Infection of leaves inoculated with VD under different treatments (**a, b, c and d**: leaf parts inoculated with VD. **e, f, g and h**: VD infection of upland cotton cotyledons) shown through a fluorescence microscope. **(C)** Average fluorescence density value of upland cotton roots. **(D)** Number of spores on upland cotton roots. The data represent the means ± SDs; n = 3. One-way ANOVA (P< 0.05) followed by Duncan’s test was performed for multiple comparisons. The different letters indicate significant differences at the 0.05 probability level. Bars = 500 µm.


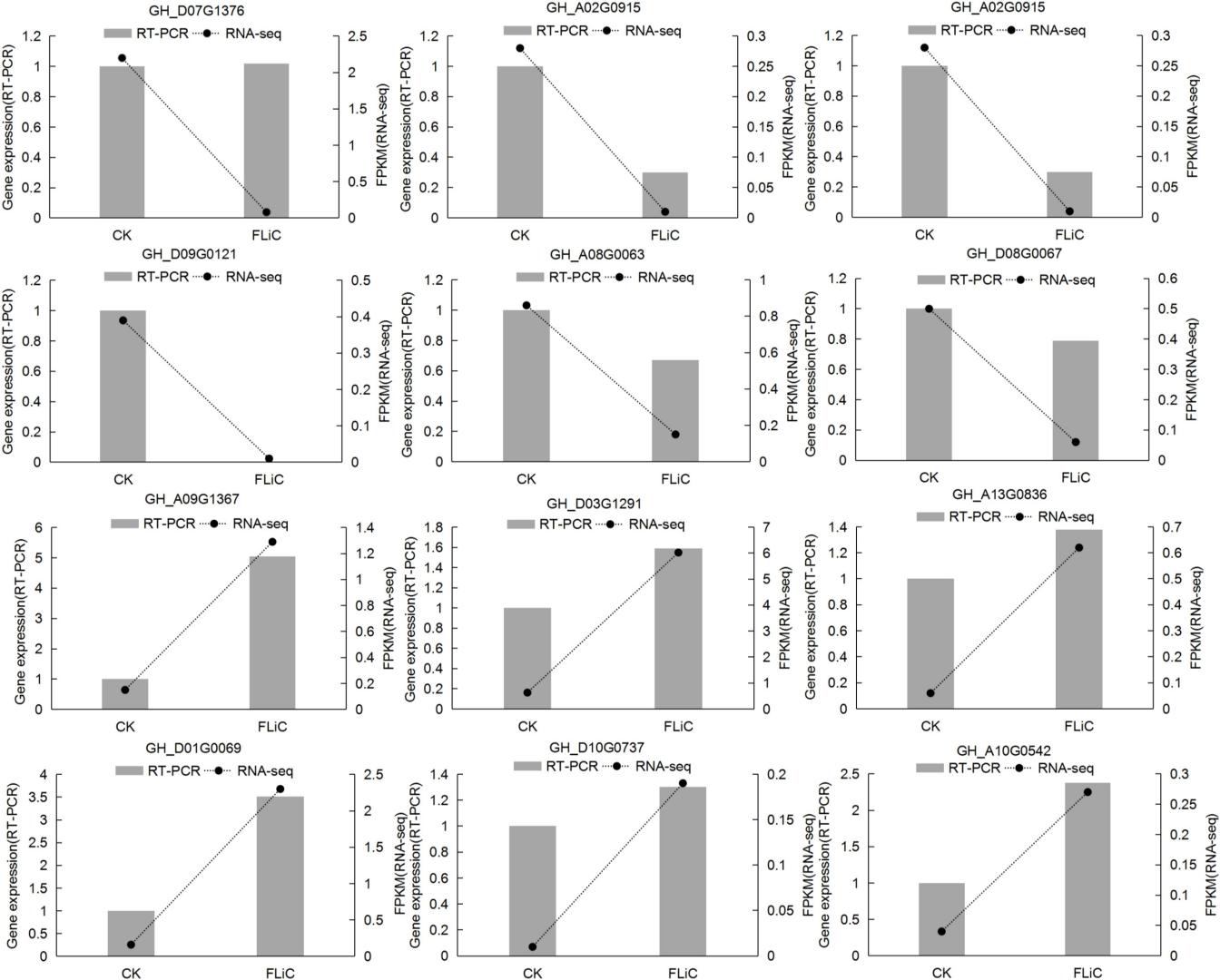


**Supplementary Figure 6** Verification of FLiC transcriptome results


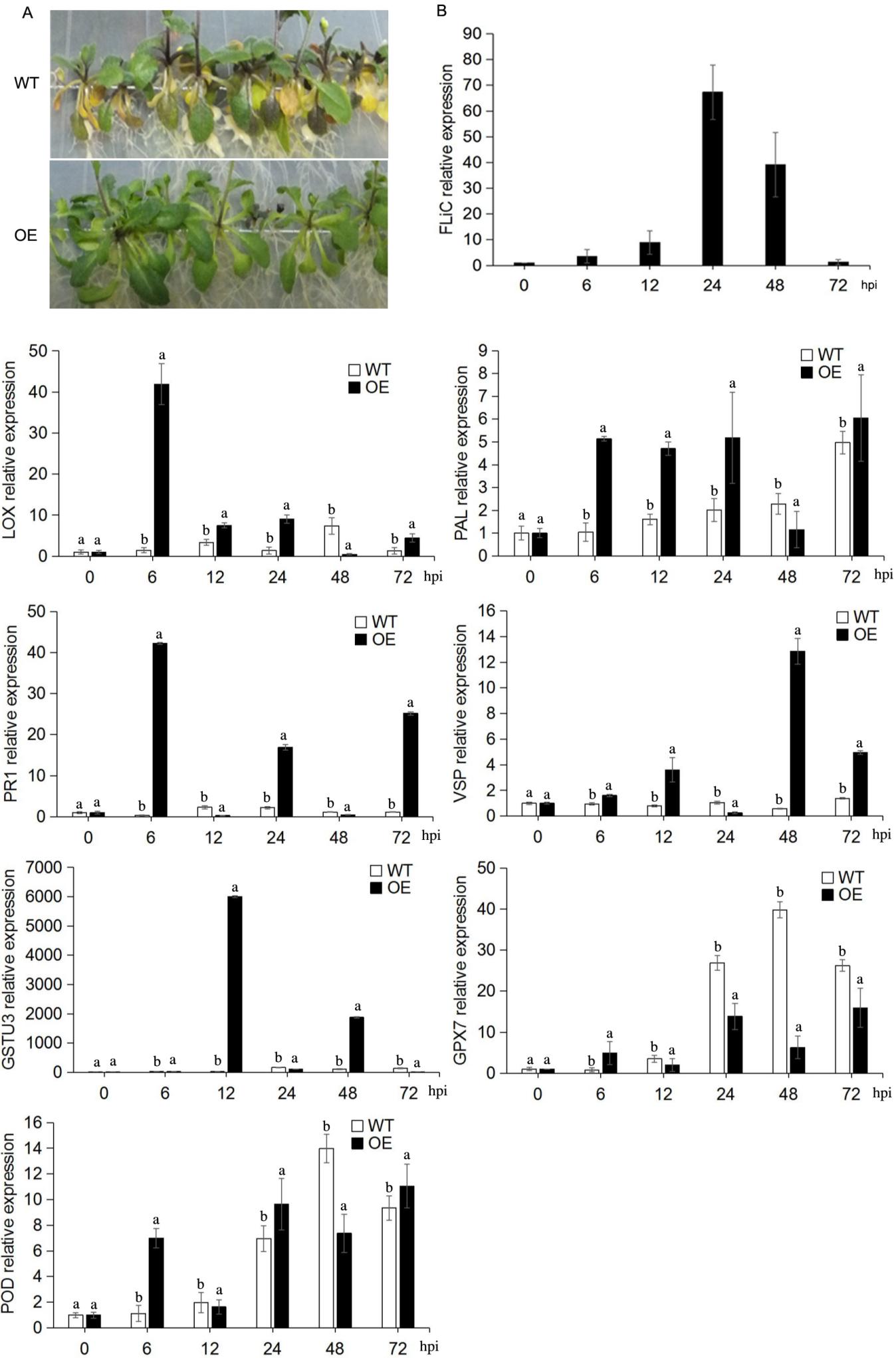


**Supplementary Figure 7** Detection of disease resistance and expression of resistance-related genes in transgenic Arabidopsis plants overexpressing *FLiC*. **(A)** Arabidopsis overexpressing the *FLiC* gene presented enhanced disease resistance. **(B)** *FLiC* expression in transgenic *Arabidopsis thaliana* increased after inoculation (WT, wild type; OE, overexpression; *PAL*, phenylalanine ammonia lyase; *PR1*, disease-related protein; *LOX*, lipoxygenase; *VSP*, vegetative storage protein; *GPX7*, glutathione peroxidase; *GSTU3*, glutathione S-transferase; *POD*, peroxidase). The data represent the means ± SDs; n = 3. The different letters indicate statistically significant differences (p < 0.05), as determined by t tests.

**Supplementary Tables**

**Supplementary Table 1** Primer sets used for qRT–PCR

| Primer | Sequence | Reference | Gene |
| --- | --- | --- | --- |
| *CHI* | ACCAAGCTACTCGCAAGAGG′  CGGAAGCGCAGTAAGATGA | CD485880 | Pathogen-induced  class 1 chitinase |
| *GLU* | CATTGATATGACCTTGATCG  GTGAGATATCCCTTGGATTG | CD486342 | Pathogen-induced  glucanase |
| *HMG* | GATTTGAAGTTGTATTTGGAG  GAAATCAGTTTGAAGGAAA | CD486522 | Pathogen-induced  HMG-CoA reductase |
| *LOX* | AGTCGTCGGTTCATGCCTGAGAAA  ATTCCCAGGAGTGTCTGCAGTTGA | Han, 2014 | Pathogen-induced  lipoxygenase |
| *POD4* | TTTGCTGCTGCCATGGTGAAGATG  CAATCAGTTGACCACCCTGCAGTT | AF155124 | Pathogen-induced  peroxidase |
| *PR1* | TGCCCAAGACTCACAACAAG  GGCCTTCTCATTAACCCACA | Han, 2014 | Putative PR1 |
| *ERF5* | GCTCAAGCCGGTTTAAATATGGGT  TTCACCACATGTACAAGGTCCCAC | Han, 2014 | Bacterium-induced ERF5 gene |
| *CDN1* | GCCAACTTGTGGTTATGCCATGCT TGCTGAGCAATCGTCTTCTCTCCT | Han, 2014 | Terpenoid aldehydes  and cadalene  derivatives |
| *PR10* | ATGATTGAAGGTCGGCCTTTAGGG  CAGCTGCCACAAACTGGTTCTCAT | Han, 2014 | Putative PR10 |
| *MAPK2* | TTACAATCTTATTCCACACACGC  TCCCTATTTATAGAAAACCTCCC | DQ132852.1 | Mitogen-activated protein kinase |
| *MAPK6* | GGACACTGAGATGGCGGAGC  ACCAATGGGCATAATAGGAG | JF727638.1 | Mitogen-activated protein kinase |
| *MAPK7* | CAGAGCAAACTATGCCGTATG  TTACAATCTTATTCCACACACGC | FJ966888.1 | Mitogen-activated protein kinase |
| *MAPK16* | GTGTTGTTTGTTCAGCGTATG  CGTAGGAGCCTGAGAAGTTTA | FJ966889.1 | Mitogen-activated protein kinase |
| *WRKY2* | TTCTTCCCATAATACCATCC  CTCGCATAAAACTGTTAGCATC | DQ864758.1 | Transcription factor |
| *WRKY3* | GGGACGAAAGTTGTCAAAGGGAA  TCGTGTTGCTGTTTCGGTTG | FJ966887.1 | Transcription factor |
| *WRKY4* | ATTGATAACAACTTACGAGGGC  TTGAGGAGCAAGGAAGGATT | JQ081265.1 | Transcription factor |
| *WRKY5* | GCAAAGGGAACCGAGATT  ATCGGGTAGGGATGCTTG | JQ081266.1 | Transcription factor |
| *WRKY6* | AAGCCAATCAAGGGTTCTCC  GTTCTTCCAAGCACCTCTCT | JQ081267.1 | Transcription factor |
| *18S rRNA* | CCATAAACGATGCCGACCAG  AGCCTTGCGACCATACTCCC | HQ658359.1 | 18S ribosomal RNA gene |

**Supplementary Table 2** Specific primer sequences of qRT–PCR-related genes

| Gene name | Primer sequence (5′→3′) | |
| --- | --- | --- |
| *GH_D07G1376* | F:GTTGGTCACGACCGAGGAG; | R:AGCCCTCTTGAAATTAGCCCC |
| *GH_D09G0121* | F:GGTCTTTATTTATTGTGACAGGTGG; | R:GCTGGTGGAATGGAAAACCG |
| *GH_A11G1348* | F:ACAGGCAGCATACTAGGTGG; | R:TCCTCTAGCTCTTGGCCCTT |
| *GH_A02G0915* | F: GCAGTCGACATCACAAAACCTC; | R:GGCCTCCACTACGGACTCTT |
| *GH_A08G0063* | F:ACCTATACCTGGCGAGTTTTG; | R:AATCCACCCAACCACAACACA |
| *GH_D08G0067* | F: ACCTATACCTGGCGAGTTTTG; | R:AATCCACCCAACCACAACACA |
| *GH_A09G1367* | F: CCGCTGGAGGTAGGAAAAGG; | R:CGATTTCTTCGCCCGGTTTC |
| *GH_D03G1291* | F: AACACTGATGCAGAAGGTAGG; | R:TGTTGCCAGGTCAAGATGTCTA |
| *GH_A13G0836* | F: CCATCTTCTCCCTCTTGGCA; | R:AAGCTGGGTTACAAGGCAAG |
| *GH_D01G0069* | F: GTGTCTGGCTTTGTCCTGGTA; | R:CTTCGATCGATGCAAACGACA |
| *GH_D10G0737* | F:ACTCCTTGTTACCAGCCGAAA; | R:CTCTGTGAAGACAAGCTGCC |
| *GH_A10G0542* | F:AGTTCGTCCAAGCGAAGGAG; | R:TCTTCAACCCCCGCTTTACC |
| *Ubiquitin* | F: GAGTCTTCGGACACCATTG; | R:CTTGACCTTCTTCTTCTTGTGC |
| FliC- Pgex-4T-2F/R | F:CGCGGATCCATGGCCTTGACCGTCAACAC | R:CCGGAATTCTTAGCGCAGCAGGCTCAGAAC |
| GhCAX3-VIGS-F/R | F:GCTCTAGACAAAGAAACATGTTG | R:CGCGGATCCTCAGTTAGGAAGCTA |
| pBI121-FLiCF/R | GCTCTAGAATTGCCTTGACCGTCAACAC | CGAGCTCACGCAGCAGGCTCAGGA |
